# New insights into island vegetation composition and species diversity—Consistent and conditional responses across contrasting insular habitats at the plot-scale

**DOI:** 10.1371/journal.pone.0200191

**Published:** 2018-07-06

**Authors:** Dirk Hattermann, Markus Bernhardt-Römermann, Annette Otte, Rolf Lutz Eckstein

**Affiliations:** 1 Institute of Landscape Ecology and Resource Management, Research Centre for Biosystems, Land Use and Nutrition (IFZ), Justus Liebig University, Giessen, Germany; 2 Institute of Ecology, Friedrich Schiller University, Jena, Germany; 3 Department of Environmental and Life Sciences – Biology, Karlstad University, Karlstad, Sweden; Ecole Pratique des Hautes Etudes, FRANCE

## Abstract

Most island-ecology studies focus on the properties of entire island communities, thus neglecting species-environment relationships operating at the habitat-level. Habitat-specific variation in the strength and sign of these relationships will conceal patterns observed on the island scale and may preclude a mechanistic interpretation of patterns and processes. Habitat-specific species-environment relationships may also depend on the descriptor of ecological communities. This paper presents a comprehensive plot-based analysis of local vegetation composition and species diversity (species richness and species evenness) of (i) rocky shore, (ii) semi-natural grassland and (iii) coniferous forest habitats in three Baltic archipelagos in Sweden. To identify differences and consistencies between habitats and descriptors, we assessed the relative contributions of the variable-sets “region”, “topography”, “soil morphology”, “soil fertility”, “soil water”, “light availability”, “distance” and “island configuration” on local vegetation composition, species richness and species evenness. We quantified the impact of “management history” on the descriptors of local grassland communities by a newly introduced grazing history index (GHI). Unlike species diversity, changes in vegetation composition were related to most of the variable-sets. The relative contributions of the variable-sets were mostly habitat-specific and strongly contingent on the descriptor involved. Within each habitat, richness and evenness were only partly affected by the same variable-sets, and if so, their relative contribution varied between diversity proxies. Across all habitats, soil variable-sets showed highly consistent effects on vegetation composition and species diversity and contributed most to the variance explained. GHI was a powerful predictor, explaining high proportions of variation in all three descriptors of grassland species communities. The proportion of unexplained variance was habitat-specific, possibly reflecting a community maturity gradient. Our results reveal that species richness alone is an incomplete representation of local species diversity. Finally, we stress the need of including habitat-based approaches when analyzing complex species-environment relationships on islands.

## Introduction

Islands world-wide are increasingly exposed to human pressure, global climate change and invasive species [1], which particularly affect insular plant communities. Associated changes of the major descriptors of plant communities, i.e. vegetation composition, species richness and species evenness, may have cascading effects on ecosystem properties [2]. However, island plant community vulnerability towards environmental changes largely depends on the descriptor involved and responses may vary idiosyncratically among habitat types [2,3]. Abundance and properties of various habitats are likely to influence species distribution patterns and consequently vegetation composition and species diversity at the island scale [4,5]. However, traditionally, many island-ecology studies focused on species communities of entire islands (e.g. [6–8]), thus omitting potential differences between various insular habitats or vegetation types. This may bias the analysis of insular plant community responses to changing environmental conditions. Previous studies on plant species richness in Northern archipelagos found large variation of species-area relationships among insular habitats, suggesting that further habitat-based studies are needed to understand species-environment relationships on islands [9,10]. Therefore, a bottom-up perspective considering different island habitats is urgently needed. This knowledge is imperative not only for biodiversity management, but also to predict possible impacts of environmental change on insular plant communities.

The archipelagos along the Swedish Baltic Coast represent highly diverse ecosystems with a rich landscape history that have only few counterparts world-wide [11]. They consist of thousands of islands, composed of contrasting habitats, highly variable environmental conditions and distinct vegetation patterns. Thus, they represent an ideal study system for habitat-based studies on vegetation composition and species diversity. During the last decades, the island habitats in the archipelagos faced fundamental changes caused by natural processes and human activities, e.g. management cessation of insular grasslands [12], seaward expansion of the coniferous forest limit [13], eutrophication of shore habitats [14] and increasing recreational use [15].

Previous studies on plant species richness in Baltic archipelagos identified several potential drivers of insular species diversity, such as island area and habitat diversity [7,10], human land use, soil heterogeneity and the surrounding landscape matrix [12,16]. In contrast, multivariate analyses on potential determinants of vegetation composition in Baltic archipelagos are scarce. Von Numers and van der Maarel [17] showed that changes in local vegetation composition in the Southwest Finnish archipelago are mainly related to environmental differences, such as island size, human impact, maritime influence and abiotic habitat conditions. Existing studies on plant-environment relationships, including those mentioned, usually concentrate on a single descriptor of plant communities, i.e. either on vegetation composition [18,19] or on species diversity, often estimated as species richness [9,20,21]. Yet, species diversity includes two complementary proxies, viz. species richness, describing the number of species in a given community, and species evenness, which reflects the similarity in species abundances in this community [22]. Few studies considered both proxies (see for example [23–26]), although one proxy alone could be a misleading indicator of species diversity [23,27]. Species richness and evenness together are related to ecosystem stability, productivity and population dynamics [27]. Thus, the present knowledge does not allow general conclusions about possibly multifaceted determinants of species richness, species evenness and vegetation composition of different habitats in complex archipelago landscapes.

This paper presents a comprehensive analysis of vegetation composition, species richness and species evenness, using a comparative approach across contrasting insular habitats. We address the obvious need for more habitat-based studies to advance the understanding of species-environment relationships on islands. We examined the relative contributions of the same sets of environmental variables on local (plot-scale) vegetation composition, species richness and species evenness in the habitats (i) rocky shore, (ii) semi-natural grassland and (iii) coniferous forest. All three are common insular habitats of Baltic archipelagos in Northern Europe. Rocky shore is a very abundant coastal habitat type, exposed to a high degree of abiotic stress and disturbance, with sparse vegetation clustered in soil-filled rock crevices. Semi-natural grassland is an open habitat type, mostly created through grassland management and dominated by grasses and herbs. Coniferous forest is a comparably stable habitat type, dominated by coniferous trees, on less disturbed sites with a closed vegetation cover. The selected habitats qualify for the present study as they perfectly reflect the whole range of insular habitat variability and ongoing environmental changes in the archipelagos. Our environmental matrix consists of ten variable groups (hereafter denoted as variable-sets), addressing the local environment (topography, soil morphology, soil fertility, soil water, vegetated area, light availability and grazing history), the surrounding landscape structure (distance and island configuration) and the effect of different archipelagos (region).

We firstly hypothesize (H1) that the effects of the environmental matrix on local vegetation composition and species diversity vary among rocky shore, semi-natural grassland and coniferous forest, i.e. are highly conditional on habitat identity.

In fragmented landscapes, local environmental conditions and the surrounding landscape structure were identified as strong predictors of vegetation composition and species diversity of local plant communities [16,28,29]. Local controls, however, are likely to exceed the importance of the landscape context at smaller scales [28]. Therefore, we secondly hypothesize (H2) that, in each of the three habitats, the relative contribution of local environmental conditions to variation in local vegetation composition and species diversity will be stronger than that of landscape structure.

Previous studies addressing vegetation composition and species diversity [28,30,31] or species richness and evenness [24,27,32] showed that these main descriptors of local plant communities may respond differently to variation in environmental conditions. Therefore, we thirdly hypothesize (H3) that, within the same habitat, variation in vegetation composition, species richness and species evenness will be related to different environmental factors.

## Materials and methods

### Study regions

**Fig 1:**
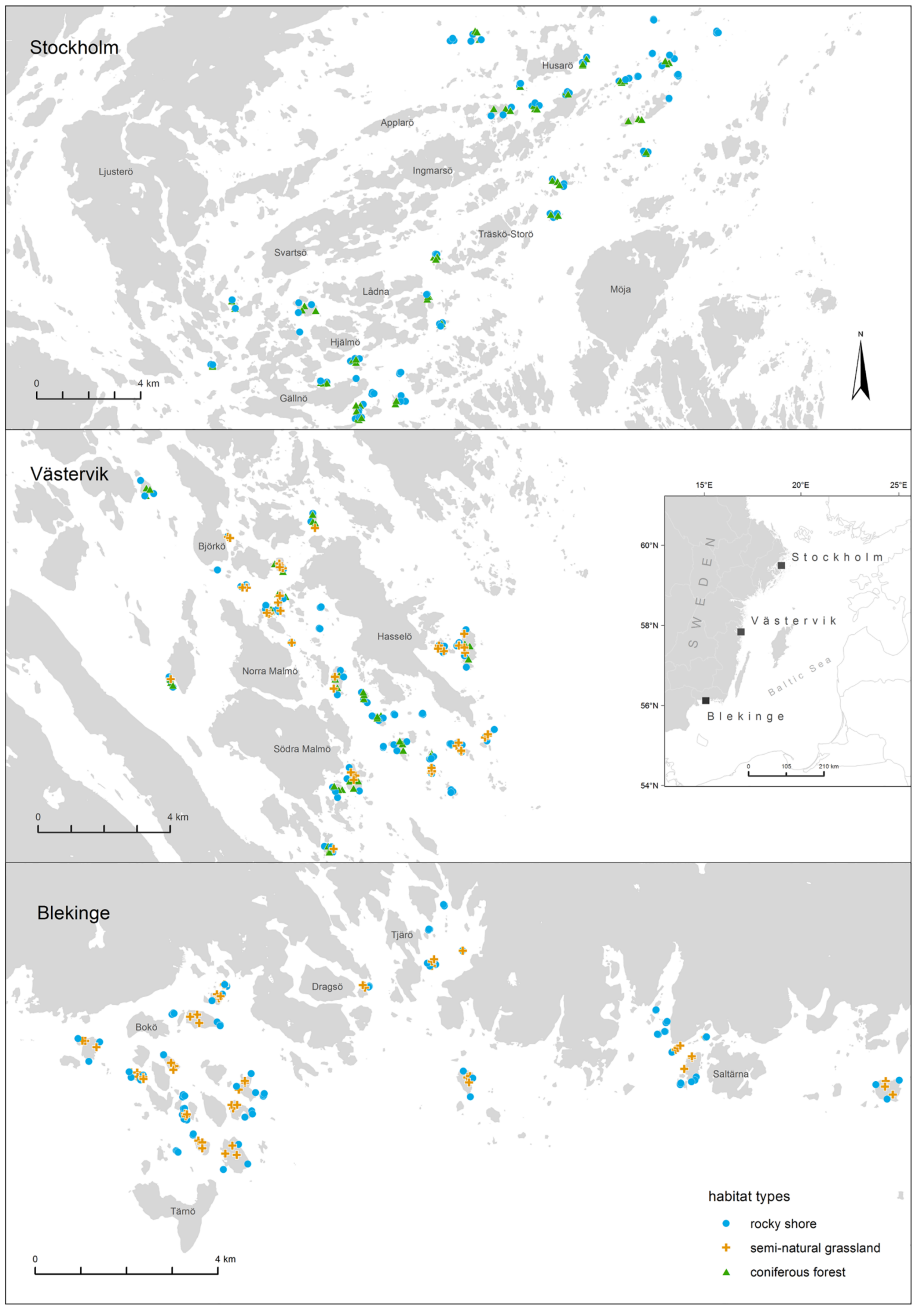
Map of the three study regions and sampling plots. Plot locations are shown were habitats were surveyed during summer 2015 and 2016 (rocky shore: N = 282; semi-natural grassland: N = 100; coniferous forest: N = 112).

We selected three study regions along the Swedish Baltic coast (Fig 1): Stockholm archipelago (59° 26’ N, 18° 43’ E), Västervik archipelago (57° 50’ N, 16° 41’ E) and Blekinge archipelago (56° 8’ N, 15° 2’ E). Each region covers approximately 400 km^2^ and comprises islands of different size, elevation and habitat composition. An indistinct gradient ranges from large forested islands near to the mainland to exposed and sparsely vegetated islets towards the open sea. Deeper water straits cut deep into the inner archipelago zones, forming maritime enclaves [33]. Most of the islands emerged from the sea during the last 3000 years as a result of post-glacial isostatic land uplift [34]. The bedrock chiefly consists of acidic siliceous rocks and the soils are mostly shallow, formed by quaternary deposits, such as morainic till, postglacial sands or glacial clays [34]. The mean annual temperature ranges from 5 °C in Stockholm to 6 °C in Västervik and Blekinge [35]. The mean annual precipitation in all study regions is approximately 600 mm year ^-1^ [35]. All study regions share a similar landscape history. For centuries, they were used for farming, fishing, and forestry. Since the first half of the 20^th^ century farming in the archipelagos has gradually ceased and most of the insular grasslands have been abandoned [7,36]. Today, many islands are occupied by summer cottages and used for recreational activities.

### Vegetation sampling, environmental variables and species diversity

We visited in total 97 islands during the growing seasons in summer 2015 and 2016. Islands occupied by houses and larger than 50 ha were not sampled, due to their mainland character and to minimize the impact of human settlements [12]. Representative sampling plots were used to be able to capture habitat-specific variation of local community structures and environmental conditions. Wherever possible, on each island, local vegetation was recorded on three standardized sampling plots within each habitat (Fig 1, Table 1). For each species, cover was recorded using the Braun-Blanquet approach [37], using the cover scale as proposed by Pfadenhauer et al. [38]. A brief description of the habitats rocky shore, semi-natural grassland and coniferous forest is given in Table 1 and a short overview of the most frequent species associated with each habitat is given in S1 Table. S2 Table contains a complete list of surveyed plant species and associated habitats. Field permissions were granted by the County Administrative Boards Stockholm and Kalmar (Västervik). For Blekinge permission was not required.

**Table 1:** Description of the insular habitats studied in the three study regions Stockholm, Västervik and Blekinge.

Habitat	Studied region	Plot N	Plot size (m)	Description	Number of taxa		
					Total	Min	Max
Coniferous forest	Stockholm, Västervik	112	10 x 10	Forest with varying dominance of *Pinus sylvestris* L. or *Picea abies* (L.) Karsten and *Juniperus communis* L. in the understorey. Crown cover > 25%, tree height > 3 m. Deciduous trees commonly intersperse. Occupies most of the islands, but gradually declines towards the open sea.	128	6	47
Semi-natural grassland	Västervik, Blekinge	100	2 x 2	Mosaic-like, nutrient-poor insular grassland with a patchy distribution. Varies from low growing, herb-rich sites on grazed grounds to species-poor communities dominated by few tall graminoids and woody species. Island grazing largely ceased during the first half of the 20^th^ century.	155	2	33
Rocky shore	Stockholm, Västervik, Blekinge	282	4 x 2	Open, sparsely vegetated coastal rocks and outcrops of the supralitoral. Exposed to infrequent seawater fluctuations and desiccation stress. Vegetation concentrated in soil-filled rock crevices (soil depth < 20 cm).	165	2	23

All environmental data presented in Table 2 were recorded on each sampling plot. We used Multispectral Satellite Personal Tracker images, supplied by the Swedish National Land Survey [39] to create a GIS island database and to compute variables related to the landscape structure. To account for edaphic effects on vegetation composition and species diversity, we took soil samples within each plot for later soil chemical analyses. For methodological details, see S3 Table. To include surrogate data on soil water, we used weighted plot mean Ellenberg indicator values for moisture [40]. Previous studies have repeatedly demonstrated the practicability of Ellenberg indicator values for Southern Scandinavia [30,41,42].

**Table 2:** Description of local (LOCAL) and landscape (LANDSCAPE) explanatory variables.

Variable-set	Variable	Description
**LOCAL**		
Topography	ELEV	Plot elevation (m.a.s.l.).
	EAST	Eastness, i.e. easterly aspect of a plot.
	NOR	Northness, i.e. northerly aspect of a plot.
	SLO	Plot slope (°).
Soil morphology	SKEL	Skeletal fraction of the soil (%).
	SOIL_D	Substrate depth to bedrock (m).
	TYPE_CL, TYPE_SN, TYPE_RO	Quaternary soil deposits: glacial clay (TYPE_CL), sandy till or post-glacial sands (TYPE_SN), rock (TYPE_RO). Soil type was not defined for the rocky shore plots.
Soil fertility	COND	Soil conductivity (uS cm^-1^).
	CN	Carbon-nitrogen ratio of the soil.
	P	Soil phosphorus (mg kg^-1^).
	PH	Soil pH.
Soil water	EIV_M	Weighted mean Ellenberg indicator value for moisture.
Light availability	OPEN	Site openness (%) of a plot.
Grazing history	GHI	Grazing history index, a relative temporal estimate of management abandonment of grassland sites. Only calculated for grassland plots.
Vegetated area	VEG_A	Vegetated area, a proxy for the plant available area in rock crevices. Only applied for rocky shore plots.
**LANDSCAPE**		
Distance	DMI	Distance to the mainland or the nearest large island ≥ 50 ha.
	PROX	Proximity index. Considers the size and proximity distance of all islands (or the mainland) within a 500 m radius.
	REI	Relative wave exposure index, based on wind frequency and speed of eight compass directions and their weighted fetch.
Island configuration	HAB_A	Habitat area.
	ISL_A	Total island area.
	R_COV	Rock cover (%) of an island.
	T_COV	Tree cover (%) of an island.

Variables of the environmental matrix were used to analyze effects on vegetation composition and species diversity in the habitats rocky shore, semi-natural grassland and coniferous forest.

We consider livestock grazing as a relevant anthropogenic driver in semi-natural unfertilized grasslands in our study regions [7,12]. To obtain an estimate of grazing history for grassland plots, we used the plant indicator system by Ekstam and Forshed [43], which is based on the species-specific response of grassland specialists to progressive succession after management abandonment. We translated their system into a numerical scale, to obtain quantitative estimates of plot-specific grazing history, here called the grazing history index GHI (for details see S4 Table and S1 Text).

Great variation of wave and wind conditions from the inner sheltered archipelago zones towards the open sea can strongly influence island plant life [44]. To include possible effects of wind and wave exposure on our species data, we computed the relative exposure index value (REI) with the Wave Exposure Model (WEMO 4.0) by Malhotra and Fonseca [45] (for details see S5 Table).

All environmental variables were grouped into the following variable-sets to generate ecologically interpretable variance components (Table 2): topography, soil morphology, soil fertility, soil water, light availability, vegetated area, grazing history, island configuration, distance and region. All variables were distinguished into local and landscape variables (cf. Table 2).

For univariate analyses of species diversity we used the two complementary proxies species richness and species evenness [32]. For species evenness we used the evenness index E_var_, proposed by Smith and Wilson [46], which describes the equality of species abundances in a community. Species evenness is independent of species richness and not biased with regard to minor and abundant species.

### Statistical analyses

For each habitat separately, all numerical explanatory variables were normalized by scaling between zero and one [47]. In case of correlated explanatory variables (r > 0.6) within each environmental matrix of the respective habitat, the variable with greater ecological significance (based on personal decision) remained in the matrix for further analyses (see S6 Table).

#### Vegetation composition

We used partial canonical correspondence analysis (pCCA) in combination with variance partitioning, to analyze the relative contributions of the explanatory variables to vegetation composition. For each habitat and variable-set we run CCA stepwise forward selection procedures with associated unrestricted Monte Carlo permutation tests (9999 permutations) (S6 Table) as implemented in CANOCO 5 [48]. Hereby, we gained reduced sets of variables best explaining the residual variation in each model [49]. Set members that did not contribute significantly to the explained variance (p ≤ 0.05, false discovery rate correction) were excluded (S6 Table). Species cover data were arcsine-square-root transformed and rare species down-weighted. For each habitat, we conducted a CCA with all significant variables to obtain information on variance explained by the full model.

We ran pCCA analyses for each habitat separately, but always included the factor region as a covariable [50] to correct for the effect of region. For the quantification of the proportion of the total variance explained (ETV) by a variable-set, the sum of canonical eigenvalues was divided by the total inertia (TI) of the species data. To be able to compare between habitats the relative contributions of the variable-sets to variation in vegetation composition, we used the proportion of the variance explained by the full model (EMV), which was obtained by dividing the sum of canonical eigenvalues of the variable-set by the sum of canonical eigenvalues of the full CCA.

#### Species diversity

To investigate possible effects of the variable-sets on species diversity, represented by species richness and species evenness, we performed a series of linear mixed-effects models (LMM) for each habitat and diversity proxy separately. In the model environment, explanatory environmental variables (fixed effects) were nested in the random factors region and island identity. We performed a model simplification through p-value-based backward selection of the least significant variables. Reduced models were validated with ANOVA-model comparison and associated chi square tests. By this procedure we obtained minimal adequate models containing the most significant terms (p ≤ 0.05) [51]. We used restricted maximum likelihood (REML) to estimate the random-effect parameters [52]. The homogeneity of variances and the assumptions for the normality of residuals were checked visually. Two outlier plots of the diversity models of the coniferous forest habitat were excluded.

We applied the R^2^-method for mixed-effects models [53], to estimate the proportion of total variance in species diversity. We computed two types of pseudo-R^2^ values, conditional R^2^ (cR^2^) and marginal R^2^ (mR^2^). The first gives an estimate of the proportion of total variance explained by the full linear model (fixed and random factors), the latter can be interpreted as the proportion of total variance explained solely by the fixed factors, i.e. the environmental variables of the fitted linear model. In the fitted model, the remaining significant variables were assigned to their predefined variable-sets (S8 Table). For each variable-set, the estimators of all remaining set members were aggregated and divided by the sum of all estimators for each fitted model, giving their relative contribution to total explained variance based on mR^2^. The relative contribution to the total explained variance (ETV) was interpreted as the importance of each remaining variable-set for explaining species richness or species evenness [54]. The difference between mR^2^ and cR^2^ was calculated to express the relative contribution of the random factors (as an approximate estimate of the effect of region) to ETV. To obtain a relative estimate for the proportion of the variance explained by the full model (EMV), we divided the variance explained by a variable-set by the variance explained by the full linear model. All statistical analyses were calculated in R (R Core Team) using the packages lmertest [55] and MuMIn [56].

## Results

### Vegetation composition

**Fig 2:**
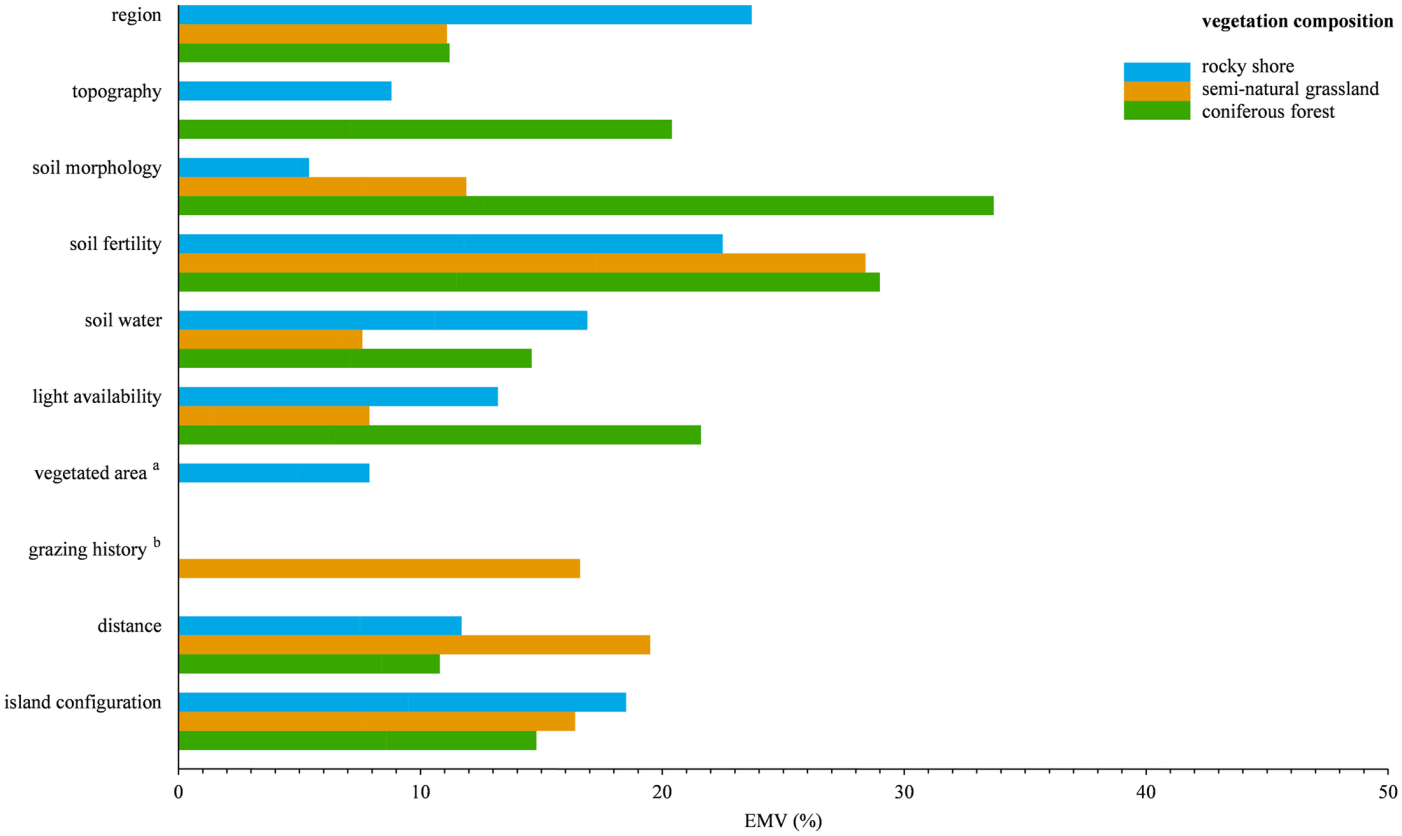
Bar chart of pCCA-based variance partitioning of vegetation composition in the studied habitats. Effects of the variable-sets and their relative contributions are shown as proportions of variance explained by the full model (EMV). Factor region was always implemented as a covariable. The effect of the variable-set region is without covariables. ^a^ analyzed only for rocky shore plots; ^b^ analyzed only for semi-natural grassland plots.

Across all habitats, local vegetation composition was highly responsive to all variable-sets (Table 3, Fig 2). The proportion of total variance explained (ETV) by the full CCA models was, however, habitat-specific. The amount of unexplained variance decreased from the highly disturbed rocky shore, via the semi-natural grassland with variable disturbance intensities, towards the relatively stable coniferous forest. The rocky shore model accounted for 18.9% ETV (TI = 4.85; F = 3.4; p ≤ 0.001), the semi-natural grassland model for 30.0% ETV (TI = 5.05; F = 2.6; p ≤ 0.001) and the coniferous forest model for 34.3% ETV (TI = 2.10; F = 3.6; p ≤ 0.001) in vegetation composition. The gradient lengths of the compositional data of the three habitats (rocky shore and semi-natural grassland = 4.3 SD; coniferous forest = 3.4 SD) indicated a higher local species turnover among rocky shore and semi-natural grassland plots than among coniferous forest plots. Except for topography, all environmental variable-sets, including region (Table 3) had significant effects on the floristic composition of all habitats. The proportions of their relative contributions in explaining vegetation composition were very similar, irrespective of whether region was implemented as the only covariable or all other variable-sets, including region, were treated as covariables (for details see S2 Fig, S7 Table and S2 Text)). For reasons of comprehensibility, the following results are based on variable-set effects with region as the only covariable.

**Table 3:** Summary statistics of the pCCA series for the three habitats.

Set	Variables	Relative contribution (% of ETV)	p-value
**Rocky shore**			
Region	REGION_B, REGION_S	4.49	≤ 0.001
Topography	ELEV,SLO	1.66	≤ 0.001
Soil morphology	SOIL_D,SKEL	1.02	0.002
Soil fertility	P, PH, COND, CN,	4.26	≤ 0.001
Soil water	EIV_M	3.20	≤ 0.001
Light availability	OPEN	2.51	≤ 0.001
Vegetated area	VEG_A	1.49	≤ 0.001
Distance	REI, DMI,	2.22	≤ 0.001
Island configuration	R_COV, ISL_A, HAB_A,	3.50	≤ 0.001
**Semi-natural grassland**			
Region	REGION_B	3.31	≤ 0.001
Topography	n.s.	n.s.	n.s.
Soil morphology	TYPE_SN, SKEL	3.57	≤ 0.001
Soil fertility	PH, CN, P	8.51	≤ 0.001
Soil water	EIV_M	2.28	≤ 0.001
Light availability	OPEN	2.37	≤ 0.001
Grazing history	GHI	6.87	≤ 0.001
Distance	PROX, REI, DMI	5.82	≤ 0.001
Island configuration	R_COV, HAB_A	4.89	≤ 0.001
**Coniferous forest**			
Region	REGION_S	3.85	≤ 0.001
Topography	ELEV,EAST	6.99	≤ 0.001
Soil morphology	TYPE_CL, TYPE_SN, SOIL_D, SKEL	11.57	≤ 0.001
Soil fertility	PH, CN	9.96	≤ 0.001
Soil water	EIV_M	5.01	≤ 0.001
Light availability	OPEN	7.41	≤ 0.001
Distance	DMI	3.69	≤ 0.001
Island configuration	ISL_A, R_COV	5.08	≤ 0.001

The relative contribution of each variable-set in explaining vegetation composition is shown. Variables are explained in Table 2. Only significant set members (p ≤ 0.05) participated in the analyses. Relevant variables in each set are presented in descending order according to their proportion of total variance explained (see CCA forward selection, in S6 Table). Relative contribution is the variance explained when controlled for factor region. Region was additionally treated as a separate set and is represented by factor levels. ETV = total explained variance; REGION_B = factor level Blekinge; REGION_S = factor level Stockholm; n.s. = set without significant variables.

The relative contribution of the factor region (Fig 2) differed between the habitats. In the rocky shore habitat, region accounted for most of the variance in floristic composition explained by the full model (23.7% EMV). The relative contribution of region on vegetation composition of semi-natural grasslands (11.1% EMV) and of coniferous forests (11.2% EMV) were almost identical, but markedly lower than its contribution on vegetation composition of rocky shores. The relative importance of single environmental variables within each variable-set partly differed between the habitats (Table 3 and S6 Table). Moreover, single environmental variables, which caused major changes in local vegetation composition in all three habitats (e.g. soil pH) (Table 3), appeared to affect the distribution of habitat-specific species rather than species shared among the habitats (see pCCA ordination graphs S1 Fig and S1 Table).

Besides region, edaphic variable-sets, i.e. soil morphology and/or soil fertility, consistently contributed most to local changes in vegetation composition in all habitats (Table 3, Fig 2). Soil fertility accounted for the largest proportion of explained variance in the rocky shore habitat (22.5% EMV) and the semi-natural grassland habitat (28.4% EMV) and had a major contribution (29.0% EMV) on compositional changes in the coniferous forest habitat. Soil morphology (33.7% EMV) exhibited the highest amount of explained variance in forest vegetation composition. As expected, soil water showed a relatively larger contribution (16.9% EMV) to variation in floristic composition of the seawater influenced rocky shore habitat than in the other, more terrestrial habitats (semi-natural grassland: 7.6% EMV; coniferous forest: 14.6% EMV).

Island configuration (Fig 2) accounted for relatively similar percentages (rocky shore: 18.5% EMV; semi-natural grassland: 16.4% EMV; coniferous forest: 14.8% EMV) of explained variance in vegetation composition in each habitat.

In all habitats, effects of light availability (cf. Fig 2) on changes in local vegetation composition were small.

Distance (19.5% EMV) (Fig 2) had a much higher impact on floristic composition in the grassland habitat, in comparison to the other two habitats (rocky shore: 11.7% EMV; coniferous forest: 10.8% EMV). Grazing history (23.0% EMV) (Table 3, Fig 2) had a major contribution to differences in vegetation composition among grassland plots on the islands, highlighting the long-term effects of continuous management as a factor selecting and promoting adapted plant species from the insular grassland species pool. Vegetated area (7.9% EMV) (Fig 2) significantly contributed to the variance explained by the full rocky shore model, underlining the effect of space limitation for certain plant species in coastal rock crevices.

In all habitats, local environment accounted for most of the variance explained by the full vegetation composition models (rocky shore: 59.4% EMV; semi-natural grassland: 66.1% EMV; coniferous forest: 72.6% EMV). When corrected for the effect of region, landscape variables (rocky shore: 28.3% EMV; semi-natural grassland: 33.5% EMV; coniferous forest: 25.5% EMV) explained less than half of the variance explained by the local environment.

### Species diversity

**Fig 3:**
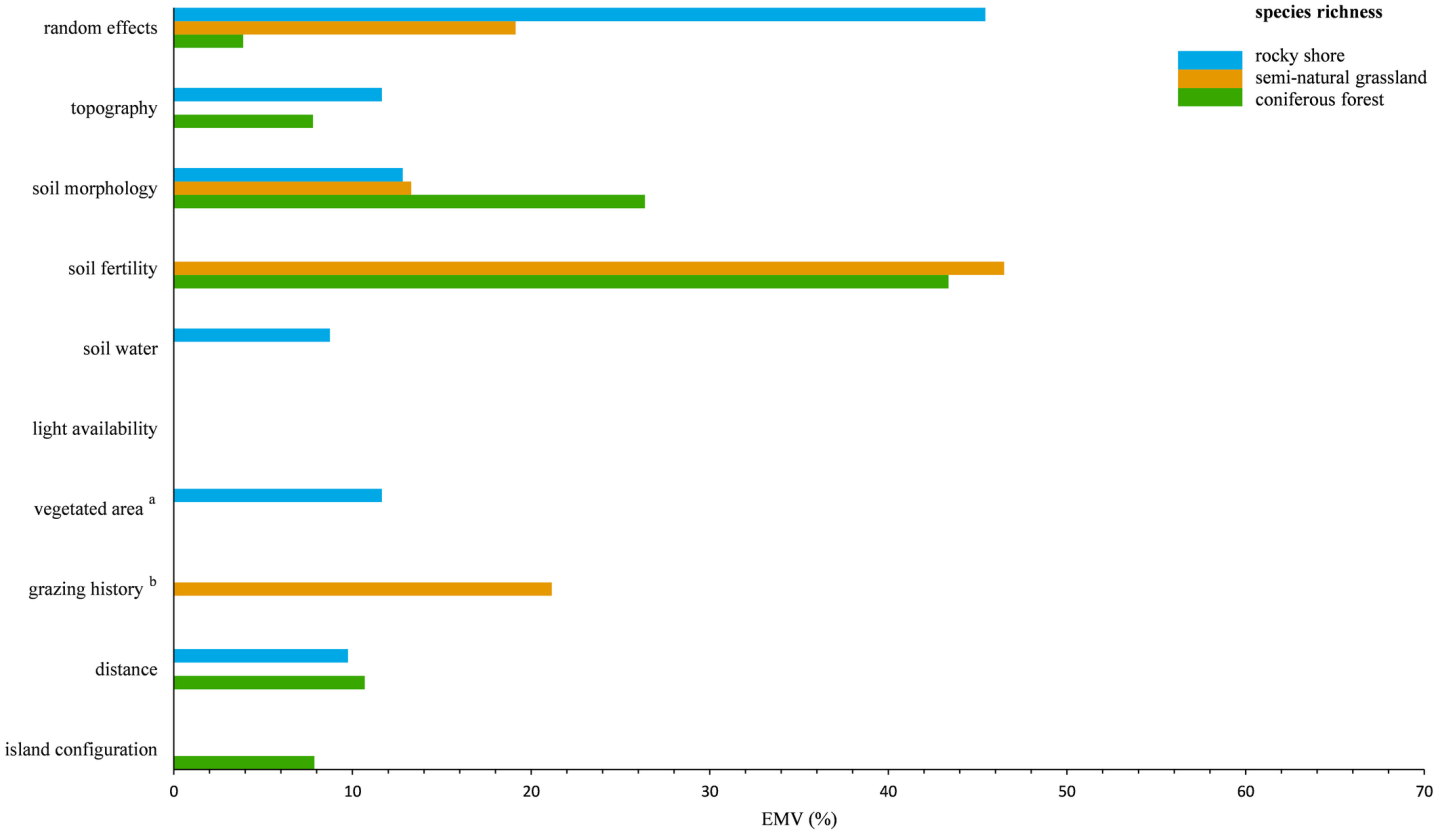
LMM-based partitioning of local species richness in the studied habitats. The relative contribution of each variable-set to the variance explained by the full model (EMV) is shown. Sets without bars did not hold significant variables (p-value ≥ 0.05) in the fitted minimal adequate LMM’s (S8 Table).^a^ analyzed only for rocky shore plots; ^b^ analyzed only for semi-natural grassland plots.

**Fig 4:**
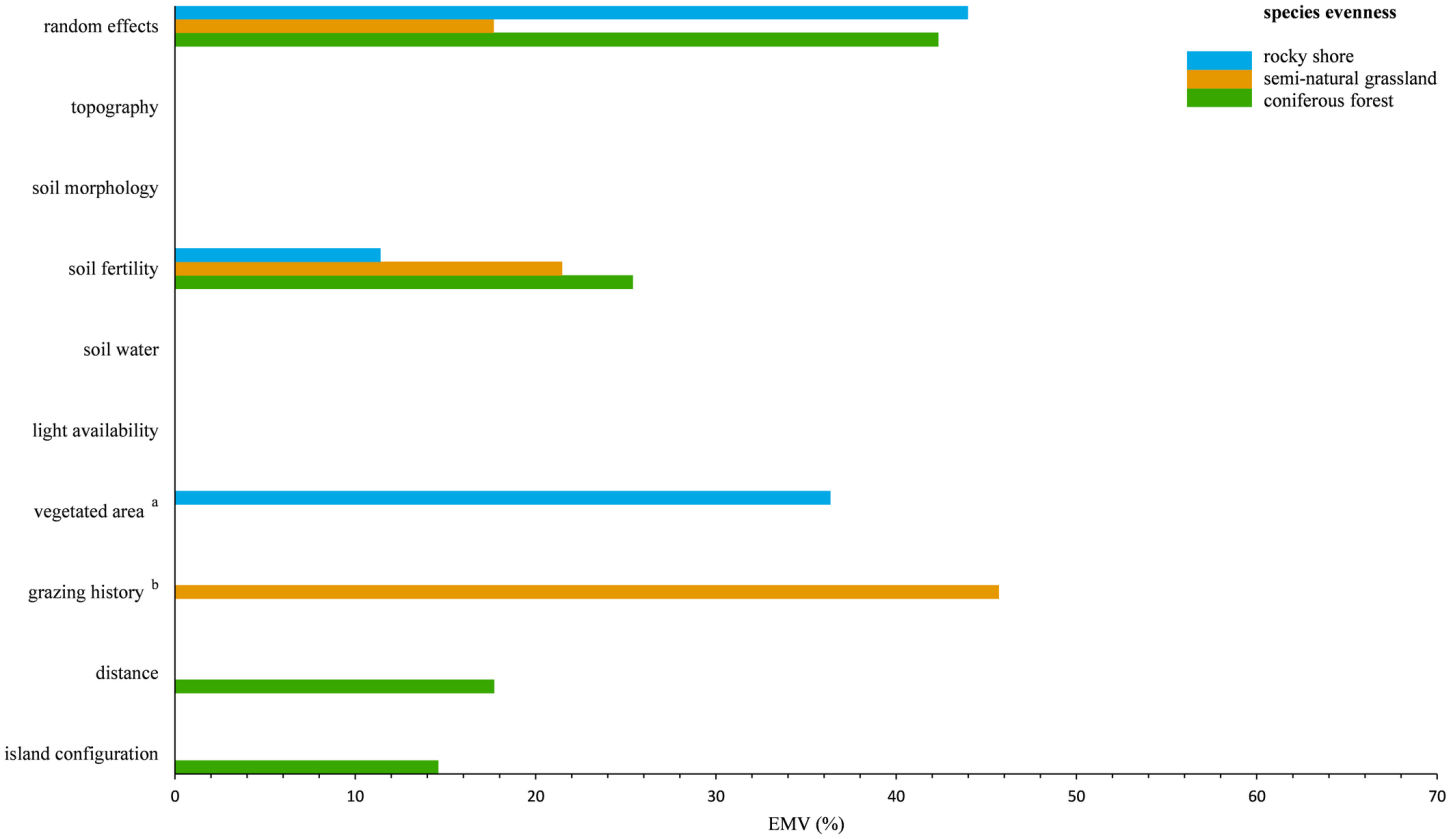
LMM-based partitioning of local species evenness in the studied habitats. The relative contribution of each variable-set to the variance explained by the full model (EMV) is shown. Sets without bars did not hold significant variables (p-value ≥ 0.05) in the fitted minimal adequate LMM’s (S8 Table). ^a^ analyzed only for rocky shore plots; ^b^ analyzed only for semi-natural grassland plots.

With a few exceptions, both the species richness models (Table 4, Fig 3) and the species evenness models (Table 4, Fig 4) revealed strong habitat-specific effects of the variable-sets on local species diversity patterns. The proportion of total variance explained (ETV) by the full LMMs varied among habitats and species diversity proxies. For local species richness, the rocky shore model accounted for 51.5% ETV, the grassland model for 75.8% ETV and the forest model for 79.9% ETV. As in the case of vegetation composition, the amount of unexplained variance decreased from rocky shore, via semi-natural grassland, to coniferous forest, possibly reflecting a community stability gradient. This trend was less pronounced for the species evenness models (rocky shore: 46.6% ETV; semi-natural grassland: 46.4% ETV; coniferous forest: 52.2% ETV).

**Table 4:** Summary statistics of LMM-based variance partitioning of local species diversity in the studied habitats.

Habitat	Diversity proxy	Variables	Set	ETV (%)
**Rocky shore**	SR	REGION, ISLAND ID	Random effects	23.42
		ELEV, SLO	Topography	6.02
		SOIL_D	Soil morphology	6.58
		EIV_M	Soil water	4.46
		VEG_A	Vegetated area	6.00
		DMI, PROX	Distance	5.02
	SE	REGION, ISLAND ID	Random effects	20.50
		P	Soil fertility	5.31
		VEG_A	Vegetated area	16.94
**Semi-natural grassland**	SR	REGION, ISLAND ID	Random effects	14.45
		TYPE_SN	Soil morphology	10.07
		PH, P, CN	Soil fertility	35.23
		GHI	Grazing history	16.04
	SE	REGION, ISLAND ID	Random effects	8.22
		PH	Soil fertility	9.96
		GHI	Grazing history	21.20
**Coniferous forest**	SR	REGION, ISLAND ID	Random effects	3.09
		ELEV	Topography	6.22
		TYPE_CL, SOIL_D	Soil morphology	21.07
		PH, CN	Soil fertility	34.65
		DMI	Distance	8.54
		R_COV	Island configuration	6.29
	SE	REGION, ISLAND ID	Random effects	22.12
		PH	Soil fertility	13.25
		DMI	Distance	9.24
		ISL_A	Island configuration	7.62

The relative contributions of each variable-set in explaining species richness (SR) and species evenness (SE) is shown. Only significant variables (fixed effects) (p-value ≤ 0.5) of the fitted minimal adequate models were evaluated and assigned as set members. Random effects were always evaluated. Variables in each set are shown in descending order, according to their proportion of total variance explained (ETV) (see S8 Table). Variables are described in Table 2.

All variable-sets, except for light availability, had a significant effect on at least one proxy of local species diversity (Table 4) in the different habitats. In general, more variable-sets explained variation in local species richness than variation in species evenness across the habitats (cf. Table 4, Figs 3 and 4). The relative importance of single environmental variables within each variable-set was only partly consistent among the three habitats and among the two diversity proxies (S8 Table). In most cases, local species richness and species evenness responded synchronously (both either negatively or positively) to single environmental variables within the variable-sets that exhibited parallel effects (cf. S8 Table). The relative contributions of the random factors region and island identity largely differed between the habitats and the diversity proxies (Figs 3 and 4). For rocky shore, random effects had the largest relative impact on both species richness (45.4% EMV), and species evenness (45.0% EMV). In contrast, random effects (richness: 19.1% EMV, evenness: 17.67% EMV) did not account for most of the variance explained by the grassland diversity models. In coniferous forests, the relative contribution of the random factors to the explained variance in species diversity was much lower for species richness (3.9% EMV) than for species evenness (42.3% EMV). The large influence of random effects on forest species dominance may be related to a high regional variability of the forest tree cover and understory characteristics, e.g. the sporadic appearance of very herb-rich, dense spruce forests in the Stockholm study region.

Partitioning variation of local species diversity revealed that most of the environmental variable-sets had strong habitat-specific effects (Table 4, Figs 3 and 4). Edaphic variable-sets had the most consistent effects on species diversity (cf. Figs 3 and 4) across all habitats. Soil fertility exhibited the largest relative contribution to variation in species richness in semi-natural grasslands (46.5% EMV) and coniferous forests (43.4% EMV). Soil morphology was also a strong predictor of grassland species richness (13.3% EMV) and forest species richness (26.4% EMV) and besides the random effects, explained most of the variance (12.8% EMV) in rocky shore species richness. Among the environmental variable-sets, soil fertility accounted for the largest proportion of explained variance (25.4% EMV) in forest species evenness, and for a considerable amount of explained variance in rocky shore species evenness (11.4% EMV) and grassland species evenness (21.5% EMV).

Apart from the parallel effects of the variable-sets topography (rocky shore: 11.65% EMV; coniferous forest: 7.8% EMV) and distance (rocky shore: 9.8% EMV; coniferous forest: 10.7% EMV) on rocky shore and coniferous forest species richness (Fig 3), the effects of all remaining variable-sets on species diversity were specific only to a single habitat (Table 4, Figs 3 and 4).

Similar to vegetation composition, local species evenness (45.7% EMV) (Fig 4) of insular grassland communities was most affected by grazing history. The latter had also a major, but comparably lower impact (21.2% EMV) (Fig 3) on grassland species richness. Altogether this underlines the importance of management history in explaining changes of local plant diversity in semi-natural grasslands. Vegetated area, as an estimate for the plant available area in rock crevices, had a major contribution (richness: 11.7% EMV; evenness: 36.4% EMV) (Figs 3 and 4) in explaining variation in local species diversity along rocky shores. Interestingly, on rocky shores, species richness was positively affected by vegetated area and species evenness was negatively affected (S8 Table).

The largest proportion of explained variance in rocky shore species diversity (richness: 44.9% EMV; evenness: 47.8% EMV), grassland species diversity (richness: 80.9% EMV; evenness: 47.8% EMV) and forest species richness (77.5% EMV) could be attributed to the local environment. In contrast, changes in forest species evenness (32.3% EMV) were rather associated with the surrounding landscape, than with the local environment (25.4% EMV). Interestingly, despite a relatively strong effect of the surrounding landscape on rocky shore and grassland vegetation composition (Fig 2), there was no landscape effect on rocky shore species evenness and grassland species diversity in general.

## Discussion

### Habitat-specific effects and contributions (H1)

We expected that the effects of the environmental matrix on local vegetation composition and species diversity vary among rocky shore, semi-natural grassland and coniferous forest, i.e. are highly conditional on habitat identity. Our results indicate that local plant communities in contrasting habitats on Baltic archipelago islands are partly affected by the same and partly by different environmental drivers and their relative contribution is mainly habitat-specific.

Unexpectedly, edaphic variables comprising soil fertility and soil morphology were highly consistent and powerful predictors of both local vegetation composition and species diversity across habitats and regions. Previous studies on vegetation composition and species diversity underpinned the central role of soil fertility and soil morphology in shaping species-environment relationships in many mainland habitats in Northern Europe [57–61]. Our study showed that these findings are also valid for plant communities of contrasting habitats within archipelago landscapes. The islands in the study regions are commonly characterized by shallow, acidic soils on solid bedrock, which could explain the overriding effects of soil pH, soil depths [58] and soil type distributions [10] on plant communities in different insular habitats. Considering the importance of single environmental variables within the variable-sets, major shifts in vegetation compositions related to soil fertility were due to changes in soil pH in all habitats. In terms of species diversity, an increase of soil pH was strongly associated with higher species numbers and more equal dominance structures in both forest and grassland communities, but this was not evident for rocky shore species diversity. Changes in soil pH can have strong effects on nutrient availability, which additionally depends on nutrient input and water availability [62] and thus on the properties and abundances of certain habitats. For example, the effect of soil fertility on composition and dominance structures of plant communities along rocky shores were largely modified by inputs of phosphorous, probably caused by sea bird droppings, partly masking existing effects of soil pH. Additional avian inputs of phosphorus and nitrogen [63–65] are known to favor nutrient-demanding and competitive plant species [65], such as *Geranium robertianum* L., *Anthriscus sylvestris* (L.) Hoffm. or *Artemisia absinthium* L., which would otherwise not be able to persist. The studied habitats share larger proportions of their species pools, since boundaries are often relatively smooth and transition zones exist. Although universal drivers, like soil pH, may determine community composition across habitat boundaries, they rather affect habitat-specific species than species shared among the habitats. Such habitat-specific species response patterns were typical for most of the consistent effects of the variable-sets on vegetation composition across the habitats. In accordance with the general predictions of island biogeographic and meta-population models, the spatial distribution of species is largely determined by the area and isolation of habitat patches in fragmented landscapes [66]. Especially species communities in patchy insular environments may be greatly influenced by colonialization processes from species pools occurring in adjacent habitats [16,66], including habitats on nearby islands and the mainland, and/or habitats from the same island. Whereas some studies in archipelago environments confirmed an effect of distance to surrounding land masses [7,67], others found distance to be a relatively weak [8,68] or no predictor [12] of insular plant diversity. Our results suggest that distance effects observed for communities of entire islands may highly depend on the presence and properties of certain habitats, on the descriptor of diversity studied and on the distance measure applied. For example, we found strong distance effects on forest species diversity, but none on grassland species diversity. Unlike most other habitats, coniferous forests show marked distributional limits towards the open sea, represented by species poor pine stands on rocky islands. This pattern may have multiple reasons, such as the very restricted dispersal abilities of forest species [69], soil type variations (i.e. the occurrence of deeper and more fertile soils on islands close to larger land masses) or harsher climatic conditions at the outer archipelago margins [13].

On the other hand, local vegetation composition in all three habitats was affected by the distance variable-set, but its relative contribution on compositional changes largely differed between the grassland habitat and the other two habitats. For example, all three complementary distance indices (relative wave exposure, proximity and distance to the mainland or large islands) contributed to compositional differences in insular grasslands, of which proximity had the strongest influence. Today, species-rich pastures, harboring a high proportion of grassland specialists, are confined to larger islands or localities in close proximity to the mainland where few farms could persist [12]. High habitat connectivity, especially to managed grassland patches on larger islands, may be vital to maintain sensitive species pools and dispersal abilities of insular grassland species in fragmented archipelago landscapes [70]. Wave exposure, as another distance proxy, had a large influence on compositional patterns of plant communities along rocky shores. Unlike interior island habitats, rock habitats exposed to the open sea are much more affected by processes such as mechanical disturbance, sea-spray, desiccation and salinization [71,72]. These conditions select for more adapted, stress-tolerant plant species, like *Allium schoenoprasum* L., *Matricaria maritima* L., and *Puccinellia capillaris* (Liljeblad) Jansen, which are able to cope with such extreme environmental fluctuations. Our findings suggest that such asymmetric, habitat-specific diversity-distance relationships need more attention when interpreting insular vegetation properties of entire islands in archipelago landscapes.

Continuous, moderate management through mowing or grazing favors high local species diversity [73], converting insular grassland patches to potential local biodiversity hotspots in the archipelagos. Historic land-use can influence present species distribution patterns, even when the management ceased long time ago [74]. In our study, grassland vegetation composition and species diversity were highly influenced by grazing history. Most of the island pastures were abandoned in the middle of the last century, they gradually overgrew and pioneer forests developed [7,36]. During this process, some grazing-dependent species were more persistent than others and could survive periods of unfavorable conditions [43]. Naturally disturbed grassland patches on open, exposed rocky islands may have the potential to support grazing-sensitive or light demanding species in the longer term. In this way, they could act as potential refuges for grassland specialists from formerly managed sites [10].

Our results imply that conservation efforts in insular grasslands should prioritize localities where considerable numbers of remnant grazing indicator species survived, or the abiotic conditions naturally support diverse plant communities. We also found that coniferous forests with the highest plant diversity are strongly confined to deep, fertile quaternary soils. In Sweden, most of the threatened forest taxa are associated with fertile soils, but ironically, such productive forests seem heavily underrepresented in Swedish protected areas [57]. Altogether, this needs more attention in the conservation management of archipelago landscapes.

In comparison to the other two, more deterministic, habitats, the rocky shore habitat was characterized by a relatively higher amount of unexplained variance and a higher species turnover. This could be attributed to the exposure of insular rock communities to strong physical disturbance [75], unpredictable stochastic events [76] and random colonialization processes [77]. In the context of island genesis [4], these habitat-specific differences in unexplained variance could be interpreted as a disturbance-driven gradient of community maturity, spanning from exposed, young island shores, with a high species turnover, via grasslands with highly variable disturbance intensities and species turnovers, towards mature, stable forest communities in the island interior. The islands in the study regions are exposed to a variety of abiotic and biotic disturbance regimes, including droughts, wind and wave action, grazing, forestry and recreational activities. Thus, habitat-specific drivers of disturbance, which act as filters on plant traits and thus on community membership [78], may play a key role in shaping local vegetation composition and species diversity on Baltic islands in particular. The question, however, to which extent habitat-specific local processes are likely to affect patterns observed on broader scales [10,79], strongly depend on the ecosystem and the spatial scales at which species communities are recorded and effects of environmental processes can be seen [79]. Altogether, our results question the common practice to interpret species-environment relationships on islands without regard to habitat-specific variation of such relationships.

### Contribution of local environment vs. landscape structure (H2)

We expected a strong contribution of the local environment in explaining changes in vegetation composition and plant species diversity. The local environment was, almost exclusively and in all habitats, a more important predictor of local vegetation composition, species richness and species evenness than the surrounding landscape structure. Relationships among local and landscape variables, however, can be complex and interactive, thus some local environmental conditions might enhance landscape effects and *vice versa* [80]. Although, the interplay between local and landscape factors seems to be highly relevant in structuring local plant communities in archipelagos landscapes [16], our results also indicate that the relative contribution of the local environment and the magnitude of the landscape context in explaining local floristic patterns are variable, depending on the habitat studied.

### Divergent responses of vegetation composition, species richness and species evenness (H3)

We hypothesized divergent responses of local vegetation composition, species richness and species evenness to the same environmental matrix within the habitats. We found that the number of potential predictors and their relative contributions in explaining changes in the response matrix differed markedly, depending on whether the focus was on vegetation composition or species diversity. This is in line with Marini et al. [28] and their findings on biotic and abiotic drivers of local plant species richness and vegetation composition of Alpine meadows. In contrast to other previous studies (e.g. [28,30]), we also found strong responses of vegetation composition and species diversity to the same variable-sets (e.g. soil properties, distance, island configuration and grazing history), but these were conditional on the habitat involved. Similar responses of vegetation composition and plant species richness to the same environmental matrix were also found by Klimek et al. [81] for managed grasslands in Germany. Species richness and species evenness are considered key descriptors of species communities [82,83]. Species richness is commonly used as a sole proxy to describe patterns of local species diversity [22,24], but our data suggest that local diversity-environment relationships largely depend on the diversity proxy involved [32]. If both proxies share important environmental drivers, such as grazing history or soil fertility in semi-natural grasslands, the strength of the relationships are proxy-specific. Generally, changes in plant species diversity are very likely to have consequences for population dynamics, ecosystem functioning and invasibility of island plant communities [1,2,84], even more so in the light of global climate change [85]. We demonstrated, that species richness alone is an incomplete representation of species diversity in complex landscapes comprising many different habitats. Thus, our study explicitly stresses the necessity to include richness and evenness as complementary proxies of species diversity[25, 86], when addressing local diversity-environment relationships on islands.

## Conclusions

We showed that a large fraction of local species-environment relationships on islands in Baltic archipelagos are strongly habitat-specific and vary with the descriptor of plant communities involved. However, the local edaphic environment was found to be a strongly consistent predictor of local vegetation composition and species diversity across three contrasting insular habitats in the study regions.

The overall effect of management history on vegetation composition and species diversity of entire islands may highly depend on the distribution and properties of management-influenced habitats in coastal archipelagos. Still, much more effort is needed to adequately assess possible impacts of environmental change (incl. management abandonment) on insular plant diversity for a wider range of island habitats. Based on our findings, we encourage further comparative studies on habitat-specific diversity-environment relationships to, firstly, better understand the habitat-specific consequences of ongoing environmental changes on insular plant communities in archipelago landscapes; to, secondly, be able to upscale these consequences to diversity patterns at the island scale; and, thirdly, to evaluate effects on the functioning and stability of insular ecosystems. Therefore, we argue for the inclusion of habitat-based approaches in future island ecology studies. Conservation efforts in Baltic archipelagos need to take into account the multitude and habitat-specificity of environmental drivers and their variable effects on different descriptors of plant communities. Finally, the impacts of habitat history and habitat disturbances need more attention when interpreting diversity-environment relationships in complex insular landscapes.

## Supporting information

S1 Dataset Raw data containing plot-based data on species cover and environmental variables. (XLSX)

S1 Fig pCCA biplot-ordination of vegetation composition of studied insular habitats. Single variables that significantly (p ≤ 0.5) contribute to compositional changes are shown as vectors (based on CCA forward selection, S6 Table)). Soil type categories (dummy variables) shown as triangle symbols. Factor region treated as covariate. 86 best fitting species are shown. For variable descriptions, see Table 2 and S3 Table. For full species names see complete species list in S2 Table. a) Rocky shore: gradient length 4.3 SD, eigenvalues axis l = 0.196 / axis ll = 0.123; b) Semi-natural grassland: gradient length 4.3 SD, eigenvalues axis l = 0.389 / axis ll = 0.172; c) Coniferous forest: gradient length 3.4 SD, eigenvalues axis l = 0.239 / axis ll = 0.146.
(PDF)

S2 Fig Bar chart of CCA-based variance partitioning of vegetation composition in the three habitat types, showing net and gross effects of variable-sets. For net effects, all other variable-sets were treated as covariates. For gross effects, the factor region was treated as the only covariable. Gross effects of region are based on CCA without covariates. Gross and net effects are presented as proportions of the variance explained by the full model (EMV). For summary statistics see S7 Table. ^a^ analyzed only for rocky shore plots; ^b^ analyzed only for semi-natural grassland plots.
(PDF)

S3 Fig Example images of the three studied insular habitats. a) Rocky shore; b) Semi-natural grassland; c) Coniferous forest.
(PDF)

S1 Table Most frequent species and species pool size of sampled habitats and proportions (%) of shared plant species between the habitats. Given percentages refer to the species pool of the habitat type in the rows, e.g. 46% of the species in the semi-natural grassland pool can be found in the coniferous forest species pool.
(PDF)

S2 Table Complete list of plant species (N = 275) surveyed in the habitats. List includes species presence (1) and absence (0) data for the sampled habitats (C = coniferous forest, G = semi-natural grassland, S = rocky shore) and species abbreviations used in the ordination plots (S1 Fig).
(PDF)

S3 Table Detailed description of environmental explanatory variables and applied methods. (PDF)

S4 Table Defined combinations of temporal-quantitative changes and associated grazing history values of pasture indicator species after management abandonment, after [1]. Category A: species strongly decline in quantity or even die out shortly after management ceased. Category B: species possibly increase during an early phase, but decrease or go extinct in the medium term. Category C: species first increase during an early and intermediate phase, but decrease in a longer term. Regression phases: T_1_ = early phase; T_2_ = intermediate phase; T_3_ = late phase. † = extinct; -2 = strong decline; -1 = moderate decline; X = unchanged; +1 = moderate increase; +2 = strong increase. All possible combinations were ranked according to an increasing regression and projected on a numeric scale ranging from 0–100, representing grazing history values.
(PDF)

S5 Table Description of data requirements and step-wise calculation of REI values. Calculated with the wave exposure model WEMO 4.0 [1] and ArcMap 10.2 (ESRI Inc., Redlands, California). REI = relative wave exposure index; SMHI = Swedish Meteorological and Hydrological Institute; IDW = inverse-distance-weighting.
(PDF)

S6 Table Summary statistics of CCA stepwise forward selection for defined variable-sets including information on collinear variables. Variables are explained in Table 2 and S3 Table. Region was treated as a separate set and is represented by factor levels. “[…]” = variable intercorrelated with variable in square brackets (r ≥ 0.6); ETV = explained total variation; “-” = variable not implemented; n.s. = not significant (p-value > 0.05); REGION_B = factor level Blekinge; REGION_S = factor level Stockholm.
(PDF)

S7 Table Summary statistics of the pCCA series for the three habitat types, showing the relative contribution of each variable-set in explaining species composition. Gross effect is the variance explained when controlled for the factor region, net effect is the variance explained when controlled for all other variables, including region. Additionally, region was treated as a separate set. ETV = total explained variance; n.s = set without significant variables.
(PDF)

S8 Table Summary statistics of the linear mixed-effects models for species richness (SR) and species evenness (SE). For each habitat and proxy of species diversity, the most significant variables (fixed effects) and implemented random effects of the fitted minimal adequate models are shown. Corresponding variable-sets are presented for the significant variables. Variables are described in Table 2 and S3 Table. Std. Err. = Standard Error; df = degrees of freedom; ETV = explained total variance.
(PDF)

S1 Text How to calculate the grazing history index (GHI). (PDF)

S2 Text Gross and net effects on vegetation composition. (PDF)
