# Supplementary material for: New insights into island vegetation composition and species diversity—Consistent and conditional responses across contrasting insular habitats at the plot-scale
Source: PLoS One. 2018 Jul 6;13(7):e0200191. doi: 10.1371/journal.pone.0200191 (PMC6034865; doi:10.1371/journal.pone.0200191)
Supplement: S1 Fig — Single variables that significantly (p ≤ 0.5) contribute to compositional changes are shown as vectors (based on CCA forward selection, S6 Table)). Soil type categories (dummy variables) shown as triangle symbols. Factor region treated as covariate. 86 best fitting species are shown. For variable descriptions, see Table 2 and S3 Table. For full species names see complete species list in S2 Table. a) Rocky shore: gradient length 4.3 SD, eigenvalues axis l = 0.196 / axis ll = 0.123; b) Semi-natural grassland: gradient length 4.3 SD, eigenvalues axis l = 0.389 / axis ll = 0.172; c) Coniferous forest: gradient length 3.4 SD, eigenvalues axis l = 0.239 / axis ll = 0.146. (PDF) [file pone.0200191.s002.pdf]

a)

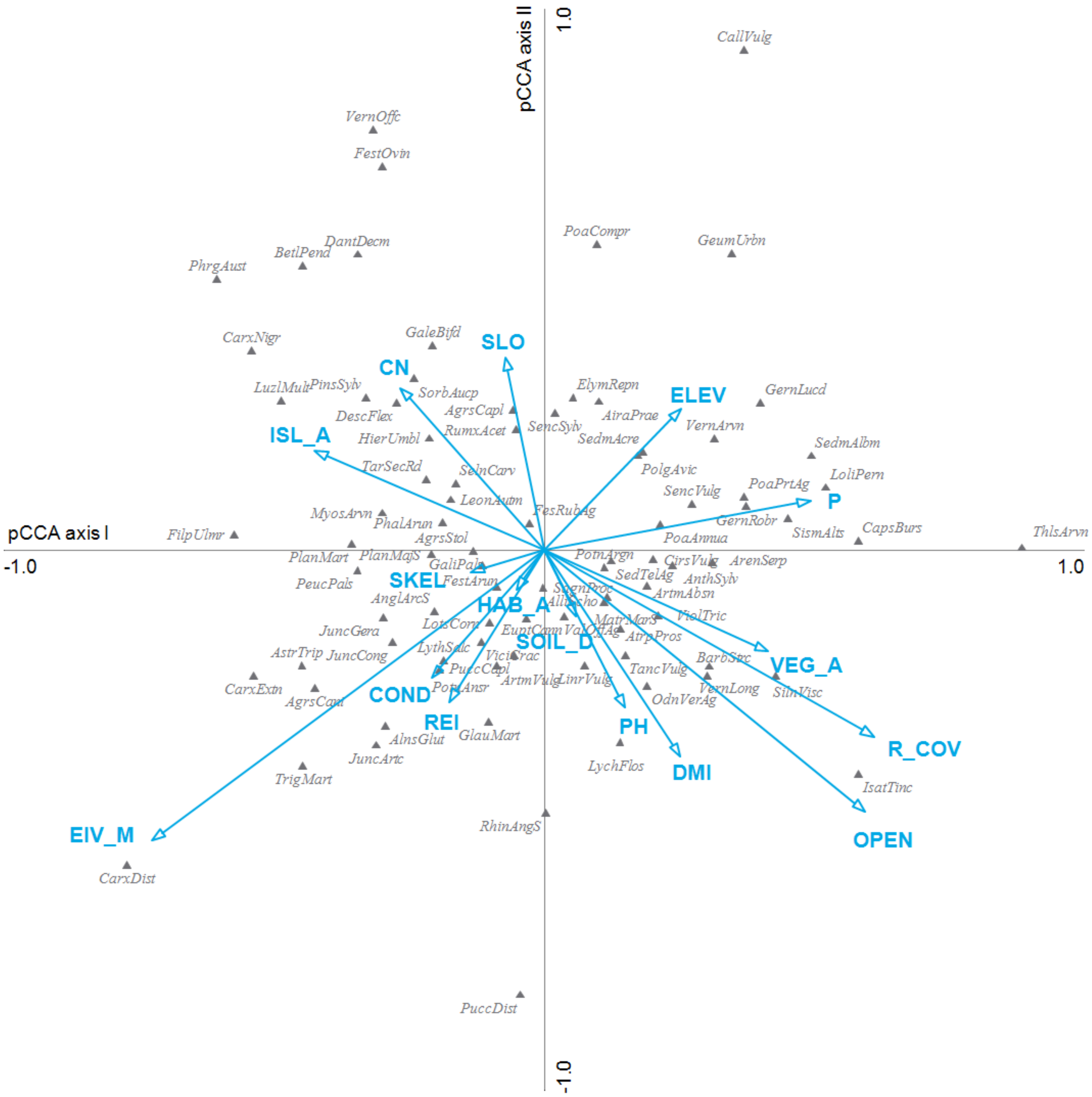

pCCA axis I
pCCA axis II
-1.0
1.0
1.0
-1.0
SLO
CN
ISL_A
ELEV
P
SKEL
HAB_A
SOIL_D
COND
REI
EIV_M
PH
DMI
VEG_A
R_COV
OPEN
CallVulg
VernOffc
FestOvin
PoaCompr
GeumUrbn
DantDecm
BetlPend
PhrgAust
CarxNigr
GaleBifd
LuzlMult
PinsSylv
SorbAucp
ElymRepn
GernLucd
AgrsCapl
AiraPrae
DescFlex
RumxAcet
SencSylv
VernArvn
HierUmbl
SedmAcre
SedmAlbm
TarSecRd
PolgAvic
LoliPern
SelnCarv
PoaPrtAg
SencVulg
LeonAutm
FesRubAg
MyosArvn
PhalArun
GernRobr
FilpUlmr
AgrsStol
SismAlts
CapsBurs
ThlsArvn
PoaAnnua
PlanMart
PlanMajS
PotnArgn
GaliPals
CirsVulg
ArenSerp
PeucPals
FestArun
SedTelAg
AnthSylv
AnglArcS
ArtmAbsn
ViolTric
LotsCorn
EuptCann
ValOffAg
MatrMarS
JuncGera
AtrpPros
AstrTrip
JuncCong
LythSalc
ViciCrac
BarbStrc
PuccCapl
TancVulg
CarxExtn
ArtmVulg
LinrVulg
PotnAnsr
VernLong
SilnVisc
AgrsCani
OdnVerAg
AlnsGlut
GlauMart
JuncArtc
LychFlos
IsatTinc
TrigMart
RhinAngS
CarxDist
PuccDist

b)

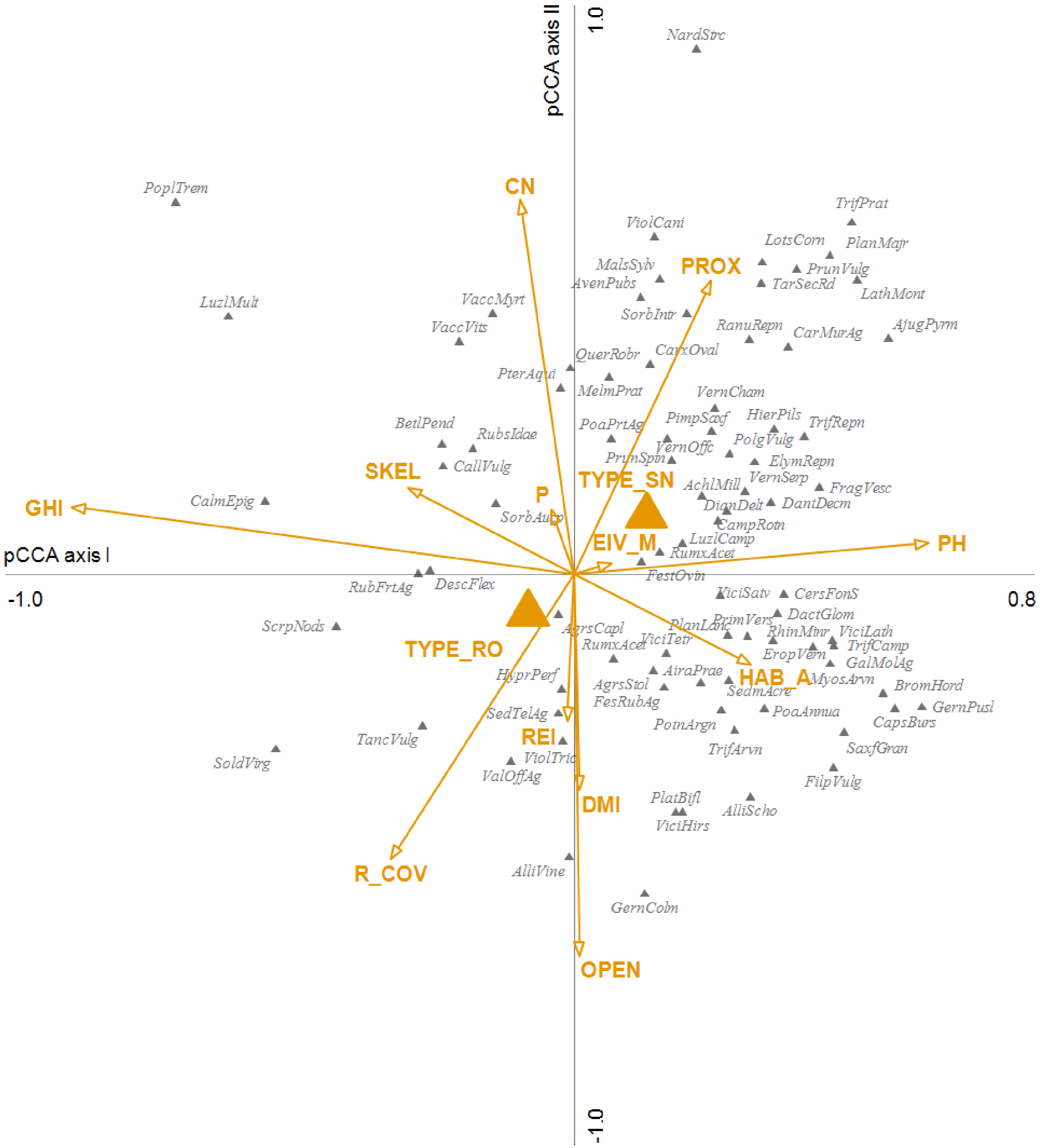

pCCA axis II
1.0
-1.0
pCCA axis I
-1.0
0.8
CN
PROX
SKEL
GHI
P
TYPE_SN
EIV_M
PH
TYPE_RO
HAB_A
REI
DMI
R_COV
OPEN
NardStrc
PoplTrem
TrifPrat
ViolCani
LotsCorn
PlanMajr
MalsSylv
PrunVulg
AvenPubs
TarSecRd
LathMont
LuzlMult
VaccMyrt
SorbIntr
RanuRepn
CarMurAg
AjugPyrm
VaccVits
QuerRobr
CarxOval
PterAqui
MelmPrat
VernCham
BetlPend
PoaPrtAg
PimpSaxf
HierPils
TrifRepn
RubsIdae
PolgVulg
CallVulg
VernOffc
PrunSpin
ElymRepn
CalmEpig
AchlMill
VernSerp
FragVesc
DianDelt
DantDecm
SorbAucp
CampRotn
LuzlCamp
RumxAcet
FestOvin
RubFrtAg
DescFlex
ViciSatv
CersFonS
ScrpNods
DactGlom
AgrsCapl
PlanLanc
PrimVers
RhinMinr
ViciLath
RumxAcet
ViciTetr
TrifCamp
EropVern
GalMolAg
AiraPrae
MyosArvn
HyprPerf
BromHord
AgrsStol
SedmAcre
FesRubAg
PoaAnnua
GernPusl
SedTelAg
CapsBurs
PotnArgn
TancVulg
TrifArvn
SaxfGran
SoldVirg
ViolTric
ValOffAg
FilpVulg
PlatBifl
AlliScho
ViciHirs
AlliVine
GernColm

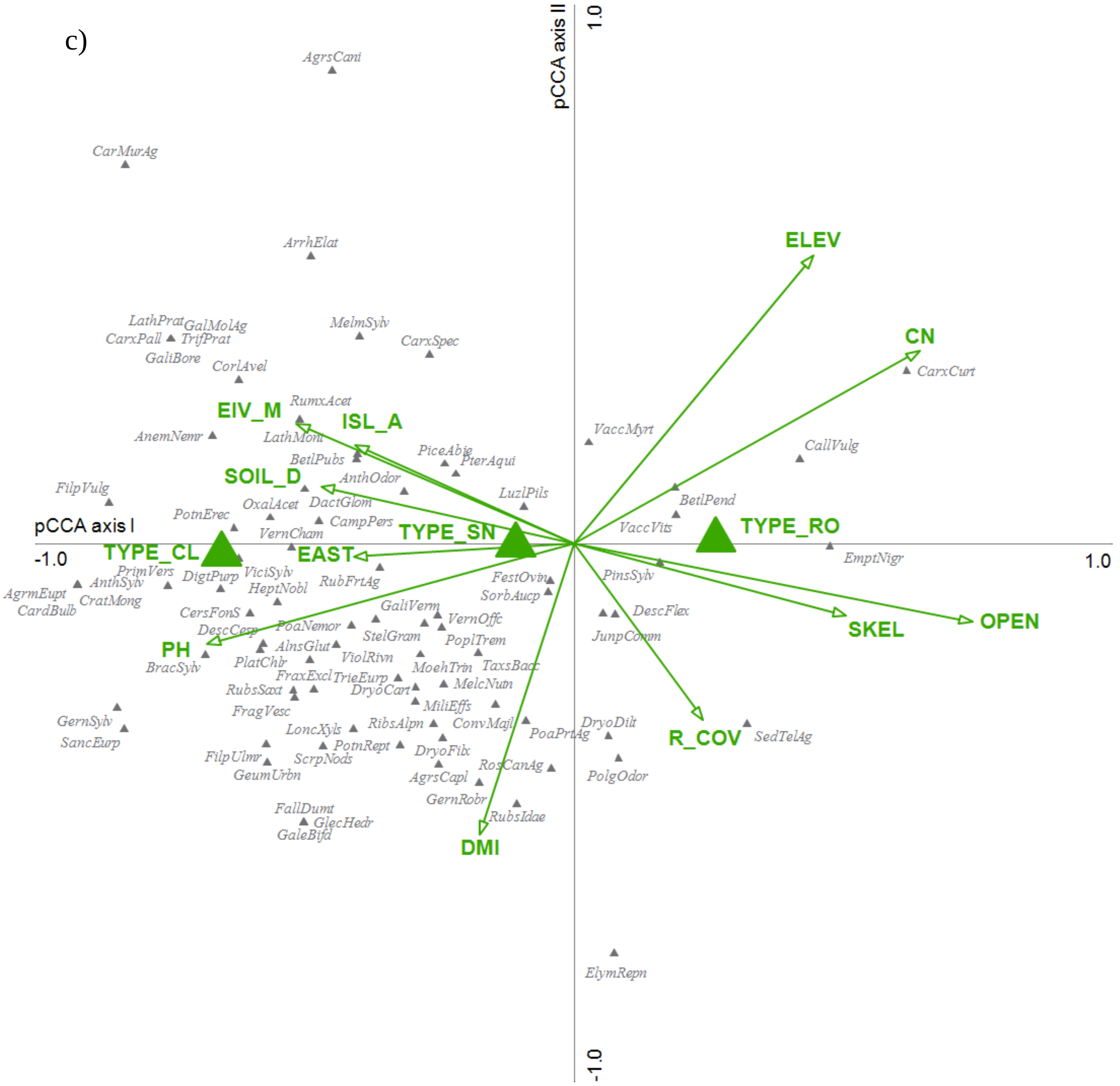

S1 Fig. pCCA biplot-ordination of vegetation composition of studied insular habitats. Single variables that significantly ($p \leq 0.5$) contribute to compositional changes are shown as vectors (based on CCA forward selection, S6 Table)). Soil type categories (dummy variables) shown as triangle symbols. Factor region treated as covariate. 86 best fitting species are shown. For variable descriptions, see Table 2 and S3 Table. For full species names see complete species list in S2 Table. a) Rocky shore: gradient length 4.3 SD, eigenvalues axis l = 0.196 / axis ll = 0.123; b) Semi-natural grassland: gradient length 4.3 SD, eigenvalues axis l = 0.389 / axis ll = 0.172; c) Coniferous forest: gradient length 3.4 SD, eigenvalues axis l = 0.239 / axis ll = 0.146.
