# Supplementary material for: New insights into island vegetation composition and species diversity—Consistent and conditional responses across contrasting insular habitats at the plot-scale
Source: PLoS One. 2018 Jul 6;13(7):e0200191. doi: 10.1371/journal.pone.0200191 (PMC6034865; doi:10.1371/journal.pone.0200191)
Supplement: S2 Fig — For net effects, all other variable-sets were treated as covariates. For gross effects, the factor region was treated as the only covariable. Gross effects of region are based on CCA without covariates. Gross and net effects are presented as proportions of the variance explained by the full model (EMV). For summary statistics see S7 Table. a analyzed only for rocky shore plots; b analyzed only for semi-natural grassland plots. (PDF) [file pone.0200191.s003.pdf]

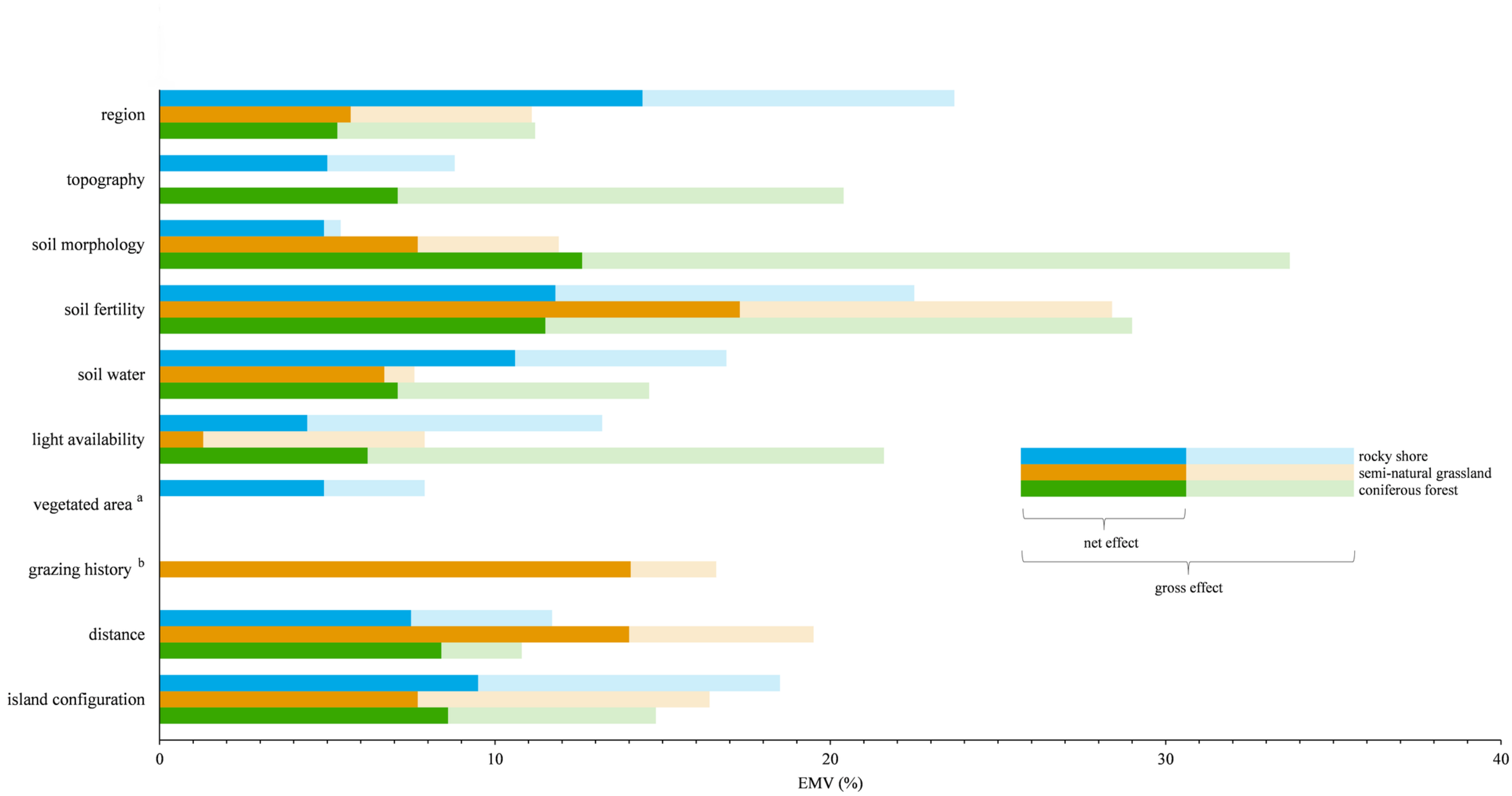

S2 Fig. Bar chart of CCA-based variance partitioning of vegetation composition in the three habitat types, showing net and gross effects variable-sets. For net effects, all other variable-sets were treated as covariates. For gross effects, the factor region was treated as the only covariable. Gross effects of region are based on CCA without covariates. Gross and net effects are presented as proportions of the variance explained by the full model (EMV). For summary statistics see S7 Table. [a] analyzed only for rocky shore plots; [b] analyzed only for semi-natural grassland plots.
