# Supplementary material for: New insights into island vegetation composition and species diversity—Consistent and conditional responses across contrasting insular habitats at the plot-scale
Source: PLoS One. 2018 Jul 6;13(7):e0200191. doi: 10.1371/journal.pone.0200191 (PMC6034865; doi:10.1371/journal.pone.0200191)
Supplement: S3 Fig — a) Rocky shore; b) Semi-natural grassland; c) Coniferous forest. (PDF) [file pone.0200191.s004.pdf]

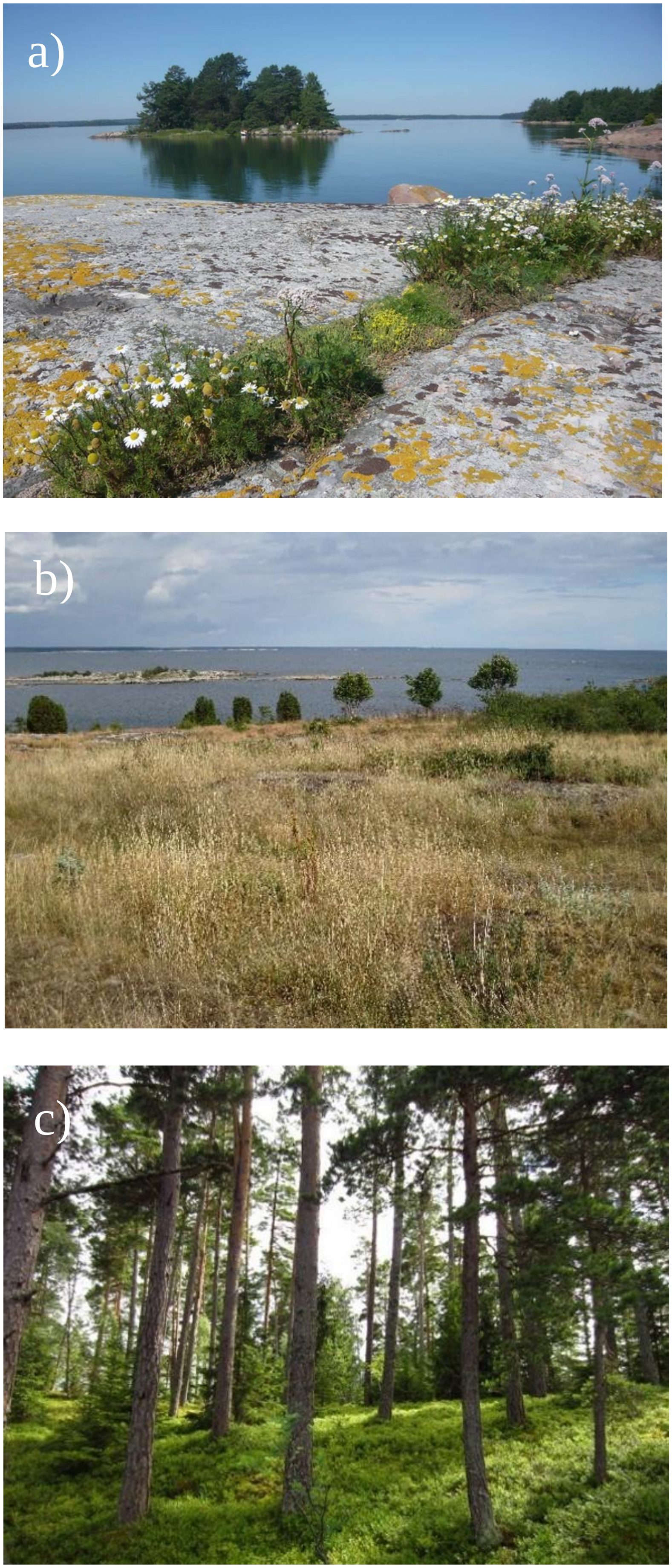

S3 Fig. Example images of the three studied insular habitats. a) Rocky shore; b) Semi-natural grassland; c) Coniferous forest.
