# Supplementary material for: New insights into island vegetation composition and species diversity—Consistent and conditional responses across contrasting insular habitats at the plot-scale
Source: PLoS One. 2018 Jul 6;13(7):e0200191. doi: 10.1371/journal.pone.0200191 (PMC6034865; doi:10.1371/journal.pone.0200191)
Supplement: S1 Table — Given percentages refer to the species pool of the habitat type in the rows, e.g. 46% of the species in the semi-natural grassland pool can be found in the coniferous forest species pool. (PDF) [file pone.0200191.s005.pdf]

S1 Table. Most frequent species and species pool size of sampled habitats and proportions (%) of shared plant species between the habitats.

Habitat type (species numbers)	Most frequent species	Coniferous forest	Semi-natural grassland	Rocky shore
Coniferous forest (128)	Tree layer: *Pinus sylvestris* L., *Picea abies* (L.) Karsten, *Betula* spp., *Populus tremula* L., *Quercus robur* L., *Sorbus* spp.<br>Herb layer: *Deschampsia flexuosa* L., *Vaccinium* spp., *Luzula* spp., *Melampyrum pratense* L., *Polypodium vulgare* L., *Pteridium aquilinum* (L.) Kuhn, *Convallaria majalis* L., *Calluna vulgaris* (L.) Hull, *Viola riviniana* Reichenb., *Poa nemoralis* L.	100	56	43
Semi-natural grassland (155)	*Deschampsia flexuosa* (L.) Trin., *Agrostis capillaris* L., *Festuca ovina* L., *Rumex acetosella* L., *Anthoxanthum odoratum* L., *Plantago lanceolata* L., *Gallium verum* L., *Achillea millefolium* L., *Festuca rubra* agg., *Luzula campestris* (L.) DC.	46	100	59
Rocky shore (165)	*Sedum acre* L., *Festuca rubra* agg., *Agrostis stolonifera* L., *Sedum telephium* L., *Leontodon autumnalis* L., *Allium schoenoprasum* L., *Matricaria maritima* L. , *Rumex crispus* L., *Sagina procumbens* L., *Valeriana officinalis* agg.	33	55	100

Given percentages refer to the species pool of the habitat type in the rows, e.g. 46 % of the species in the semi-natural grassland pool are shared with the coniferous forest species pool.
