# Supplementary material for: New insights into island vegetation composition and species diversity—Consistent and conditional responses across contrasting insular habitats at the plot-scale
Source: PLoS One. 2018 Jul 6;13(7):e0200191. doi: 10.1371/journal.pone.0200191 (PMC6034865; doi:10.1371/journal.pone.0200191)
Supplement: S2 Table — List includes species presence (1) and absence (0) data for the sampled habitats (C = coniferous forest, G = semi-natural grassland, S = rocky shore) and species abbreviations used in the ordination plots (S1 Fig). (PDF) [file pone.0200191.s006.pdf]

S2 Table. Complete list of plant species (N = 275) surveyed in the habitats.

Species name (alphabetical)	Species name and author	Abbreviated	C	G	S
Achillea millefolium	*Achillea millefolium* L.	AchlMill	1	1	1
Agrimonia eupatoria	*Agrimonia eupatoria* L.	AgrmEupt	1	0	0
Agrostis canina	*Agrostis canina* L.	AgrsCani	1	0	1
Agrostis capillaris	*Agrostis capillaris* L.	AgrsCapl	1	1	1
Agrostis stolonifera	*Agrostis stolonifera* L.	AgrsStol	0	1	1
Aira praecox	*Aira praecox* L.	AiraPrae	0	1	1
Ajuga pyramidalis	*Ajuga pyramidalis* L.	AjugPyrm	0	1	0
Allium oleraceum	*Allium oleraceum* L.	AlliOler	0	1	1
Allium schoenoprasum	*Allium schoenoprasum* L.	AlliScho	0	1	1
Allium scorodoprasum	*Allium scorodoprasum* L. s. str.	AlliScor	0	0	1
Allium vineale	*Allium vineale* L. s. str.	AlliVine	0	1	1
Alnus glutinosa	*Alnus glutinosa* (L.) Gaertn.	AlnsGlut	1	0	1
Alopecurus geniculatus	*Alopecurus geniculatus* L.	AlopGenc	0	0	1
Anemone nemorosa	*Anemone nemorosa* L.	AnemNemr	1	0	0
Angelica archangelica spp. *litoralis*	*Angelica archangelica* spp. *litoralis* (Wahlenb.) Thell.	AnglArcS	1	1	1
Anthoxanthum odoratum	*Anthoxanthum odoratum* L. s. str.	AnthOdor	1	1	1
Anthriscus sylvestris	*Anthriscus sylvestris* (L.) Hoffm.	AnthSylv	1	1	1
Arenaria serpyllifolia	*Arenaria serpyllifolia* L. s. l.	ArenSerp	0	1	1
Armeria maritima	*Armeria maritima* (Mill.) Willd. s. l.	ArmrMart	0	1	1
Arrhenatherum elatius	*Arrhenatherum elatius* (L.) P. Beauv. ex J. Presl & C. Presl	ArrhElat	1	1	1
Artemisia absinthium	*Artemisia absinthium* L.	ArtmAbsn	0	1	1
Artemisia vulgaris	*Artemisia vulgaris* L.	ArtmVulg	0	0	1
Aster tripolium	*Aster tripolium* L.	AstrTrip	0	0	1
Atriplex glabriuscula	*Atriplex glabriuscula* Edmondston	AtrpGlab	0	0	1
Atriplex prostrata	*Atriplex prostrata* Boucher ex DC.	AtrpPros	0	0	1
Avenula pubescens	*Avenula pubescens* (Huds.) Dumort.	AvenPubs	0	1	0
Barbarea stricta	*Barbarea stricta* Andrz.	BarbStrc	0	0	1
Berberis vulgaris	*Berberis vulgaris* L.	BerbVulg	1	0	0
Berteroa incana	*Berteroa incana* (L.) DC.	BertIncn	0	1	0
Betula pendula	*Betula pendula* Roth	BetlPend	1	1	1
Betula pubescens	*Betula pubescens* Ehrh. s. l.	BetlPubs	1	0	0
Brachypodium sylvaticum	*Brachypodium sylvaticum* (Huds.) P. Beauv.	BracSylv	1	0	0
Briza media	*Briza media* L.	BrizMedi	0	0	0
Bromus hordeaceus	*Bromus hordeaceus* L.	BromHord	0	1	1
Calamagrostis epigejos	*Calamagrostis epigejos* (L.) Roth	CalmEpig	1	1	1
Calluna vulgaris	*Calluna vulgaris* (L.) Hull	CallVulg	1	1	1
Calystegia sepium	*Calystegia sepium* (L.) R. Br.	CalsSepi	0	1	0
Campanula persicifolia	*Campanula persicifolia* L.	CampPers	1	0	0
Campanula rotundifolia	*Campanula rotundifolia* L. s. str.	CampRotn	1	1	0
Capsella bursa-pastoris	*Capsella bursa-pastoris* (L.) Med.	CapsBurs	0	1	1
Cardamine bulbifera	*Cardamine bulbifera* (L.) Crantz	CardBulb	1	0	0
Carex caryophyllea	*Carex caryophyllea* Latourr.	CarxCary	0	1	0
Carex curta	*Carex curta* Gooden.	CarxCurt	1	0	1
Carex distans	*Carex distans* L.	CarxDist	0	0	1
Carex extensa	*Carex extensa* Good.	CarxExtn	0	0	1
Carex hirta	*Carex hirta* L.	CarxHirt	0	1	0
Carex muricata agg.		CarMurAg	1	1	0
Carex nigra	*Carex nigra* (L.) Reichard	CarxNigr	1	1	1
Carex ovalis	*Carex ovalis* Good.	CarxOval	1	1	0
Carex pallescens	*Carex pallescens* L.	CarxPall	1	0	0
Carex panicea	*Carex panicea* L.	CarxPanc	0	0	0
Carex pilulifera	*Carex pilulifera* L.	CarxPilu	1	1	0
Carex species		CarxSpec	1	0	0
Centaurea jacea	*Centaurea jacea* L. s. l.	CentJace	0	1	1
Centaurium littorale	*Centaurium littorale* (Turner) Gilm.	CentLitt	0	0	1
Centaurium pulchellum	*Centaurium pulchellum* (Sw.) Druce	CentPulc	0	0	1

Cerastium fontanum spp. *vulgare*	*Cerastium fontanum* spp. *vulgare* (Hartman) Greuter & Burdet	CersFonS	1	1	1
Chenopodium glaucum	*Chenopodium glaucum* L.	ChenGlau	0	0	1
Cirsium arvense	*Cirsium arvense* (L.) Scop.	CirsArvn	0	1	1
Cirsium vulgare	*Cirsium vulgare* (Savi) Ten.	CirsVulg	0	1	1
Cochlearia danica	*Cochlearia danica* L.	CochDanc	0	0	1
Cochlearia officinalis	*Cochlearia officinalis* L. s. str.	CochOffc	0	0	1
Convallaria majalis	*Convallaria majalis* L.	ConvMajl	1	1	0
Corylus avellana	*Corylus avellana* L.	CorlAvel	1	0	0
Crataegus monogyna	*Crataegus monogyna* Jacq. s. l.	CratMong	1	1	1
Cuscuta europaea	*Cuscuta europaea* L.	CuscEurp	0	0	1
Dactylis glomerata	*Dactylis glomerata* L. s. str.	DactGlom	1	1	1
Danthonia decumbens	*Danthonia decumbens* (L.) DC.	DantDecm	0	1	1
Deschampsia cespitosa	*Deschampsia cespitosa* (L.) P. Beauv. s. str.	DescCesp	1	0	0
Deschampsia flexuosa	*Deschampsia flexuosa* (L.) Trin.	DescFlex	1	1	1
Descurainia sophia	*Descurainia sophia* (L.) Webb ex Prantl	DescSoph	0	0	1
Dianthus deltoides	*Dianthus deltoides* L.	DianDelt	0	1	0
Digitalis purpurea	*Digitalis purpurea* L.	DigtPurp	1	0	0
Dryopteris carthusiana	*Dryopteris carthusiana* (Vill.) H. P. Fuchs	DryoCart	1	1	0
Dryopteris dilatata	*Dryopteris dilatata (Hoffm.)* A. Gray	DryoDilt	1	0	0
Dryopteris filix-mas	*Dryopteris filix-mas* (L.) Schott	DryoFilx	1	0	0
Elymus repens	*Elymus repens* (L.) Gould	ElymRepn	1	1	1
Empetrum nigrum	*Empetrum nigrum* L. s. str.	EmptNigr	1	0	0
Epilobium angustifolium	*Epilobium angustifolium* L.	EpilAngs	0	1	0
Epilobium ciliatum	*Epilobium ciliatum* Raf.	EpilCili	0	0	1
Equisetum arvense	*Equisetum arvense* L.	EquiArvn	1	0	0
Erophila verna	*Erophila verna* (L.) Chevall. s. l.	EropVern	0	1	1
Eupatorium cannabinum	*Eupatorium cannabinum* L.	EuptCann	0	1	1
Fallopia dumetorum	*Fallopia dumetorum* (L.) Holub	FallDumt	1	0	1
Festuca arundinacea	*Festuca arundinacea* Schreb.	FestArun	0	0	1
Festuca ovina	*Festuca ovina* L. s. str.	FestOvin	1	1	1
Festuca pratensis	*Festuca pratensis* Huds. s. l.	FestPrat	0	1	0
Festuca rubra agg.		FesRubAg	1	1	1
Filipendula ulmaria	*Filipendula ulmaria* (L.) Maxim.	FilpUlmr	1	0	1
Filipendula vulgaris	*Filipendula vulgaris* Moench	FilpVulg	1	1	0
Fragaria vesca	*Fragaria vesca* L.	FragVesc	1	1	1
Frangula alnus	*Frangula alnus* Mill.	FranAlns	1	0	0
Fraxinus excelsior	*Fraxinus excelsior* L.	FraxExcl	1	1	1
Galeopsis bifida	*Galeopsis bifida* Boenn.	GaleBifd	1	0	1
Galium aparine	*Galium aparine* L.	GaliApar	0	1	0
Galium boreale	*Galium boreale* L.	GaliBore	1	0	0
Galium mollugo agg.		GalMolAg	1	1	1
Galium palustre	*Galium palustre* L. s. l.	GaliPals	1	0	1
Galium verum	*Galium verum* L. s. str.	GaliVerm	1	1	1
Geranium columbinum	*Geranium columbinum* L.	GernColm	0	1	1
Geranium lucidum	*Geranium lucidum* L.	GernLucd	1	0	1
Geranium molle	*Geranium molle* L.	GernMoll	0	1	1
Geranium pusillum	*Geranium pusillum* Burm. f.	GernPusl	0	1	1
Geranium robertianum	*Geranium robertianum* L. s. str.	GernRobr	1	0	1
Geranium sanguineum	*Geranium sanguineum* L.	GernSang	1	0	1
Geranium sylvaticum	*Geranium sylvaticum* L.	GernSylv	1	0	0
Geum urbanum	*Geum urbanum* L.	GeumUrbn	1	0	1
Glaux maritima	*Glaux maritima* L.	GlauMart	0	0	1
Glechoma hederacea	*Glechoma hederacea* L.	GlecHedr	1	1	0
Hepatica nobilis	*Hepatica nobilis* Schreb.	HeptNobl	1	0	0
Herniaria glabra	*Herniaria glabra* L.	HernGlab	0	1	0
Hieracium pilosella	*Hieracium pilosella* L.	HierPils	0	1	1
Hieracium sect. *Vulgata*		HieSecVl	1	0	1
Hieracium umbellatum	*Hieracium umbellatum* L.	HierUmbl	1	1	1
Holcus lanatus	*Holcus lanatus* L.	HolcLant	0	1	1
Hypericum humifusum	*Hypericum humifusum* L.	HyprHumf	0	1	0

Hypericum perforatum	*Hypericum perforatum* L.	HyprPerf	1	1	1
Hypochoeris radicata	*Hypochoeris radicata* L.	HypcRadc	0	1	0
Impatiens parviflora	*Impatiens parviflora* DC.	ImptParv	1	0	0
Isatis tinctoria	*Isatis tinctoria* L.	IsatTinc	0	0	1
Jasione montana	*Jasione montana* L.	JasiMont	0	1	0
Juncus articulatus	*Juncus articulatus* L.	JuncArtc	0	0	1
Juncus bufonius	*Juncus bufonius* L.	JuncBufn	0	0	1
Juncus conglomeratus	*Juncus conglomeratus* L.	JuncCong	0	0	1
Juncus effusus	*Juncus effusus* L.	JuncEffs	1	0	1
Juncus gerardi	*Juncus gerardii* Loisel.	JuncGera	0	0	1
Juniperus communis	*Juniperus communis* L. s. str.	JunpComm	1	1	0
Lathyrus montanus	*Lathyrus montanus* Bernh.	LathMont	1	1	0
Lathyrus pratensis	*Lathyrus pratensis* L.	LathPrat	1	1	0
Ledum palustre	*Ledum palustre* L.	LedmPals	1	0	0
Leontodon autumnalis	*Leontodon autumnalis* L.	LeonAutm	0	1	1
Lepidium latifolium	*Lepidium latifolium* L.	LepdLatf	0	0	1
Leucanthemum vulgare	*Leucanthemum vulgare* Lam. s. str.	LeucVulg	0	1	0
Linaria vulgaris	*Linaria vulgaris* Mill.	LinrVulg	0	1	1
Lolium perenne	*Lolium perenne* L.	LoliPern	0	0	1
Lonicera periclymenum	*Lonicera periclymenum* L.	LoncPerc	1	1	0
Lonicera xylosteum	*Lonicera xylosteum* L.	LoncXyls	1	0	0
Lotus corniculatus	*Lotus corniculatus* L.	LotsCorn	0	1	1
Luzula campestris	*Luzula campestris* (L.) DC.	LuzlCamp	0	1	1
Luzula multiflora	*Luzula multiflora* (Ehrh.) Lej. s. str.	LuzlMult	1	1	1
Luzula pilosa	*Luzula pilosa* (L.) Willd.	LuzlPils	1	1	0
Lychnis flos-cuculi	*Lychnis flos-cuculi* L.	LychFlos	0	0	1
Lychnis viscaria	*Lychnis viscaria* L.	LychVisc	0	1	0
Lycopus europaeus	*Lycopus europaeus* L.	LycpEurp	0	0	1
Lythrum portula	*Lythrum portula* (L.) D.A.Webb	LythPort	0	0	1
Lythrum salicaria	*Lythrum salicaria* L.	LythSalc	0	0	1
Maianthemum bifolium	*Maianthemum bifolium* (L.) F. W. Schmidt	MaiaBifl	1	0	0
Malus sylvestris	*Malus sylvestris* Mill.	MalsSylv	1	1	0
Matricaria maritima spp. *maritima*	*Matricaria maritima* spp. *maritima* (L.) W. D. J. Koch	MatrMarS	0	0	1
Medicago lupulina	*Medicago lupulina* L.	MedcLupl	0	1	0
Melampyrum pratense	*Melampyrum pratense* L.	MelmPrat	1	1	1
Melampyrum sylvaticum	*Melampyrum sylvaticum* L.	MelmSylv	1	0	0
Melica nutans	*Melica nutans* L.	MelcNutn	1	0	0
Milium effusum	*Milium effusum* L.	MiliEffs	1	0	0
Moehringia trinervia	*Moehringia trinervia* (L.) Clairv.	MoehTrin	1	0	0
Molinia caerulea	*Molinia caerulea* (L.) Moench s. str.	MolnCaer	0	1	1
Monotropa hypopitys	*Monotropa hypopitys* L. s. str.	MontHypo	1	0	0
Mycelis muralis	*Mycelis muralis* (L.) Dumort.	MyclMurl	1	0	0
Myosotis arvensis	*Myosotis arvensis* (L.) Hill	MyosArvn	0	1	1
Nardus stricta	*Nardus stricta* L.	NardStrc	1	1	0
Odontites verna agg.		OdnVerAg	0	0	1
Ophioglossum vulgatum	*Ophioglossum vulgatum* L.	OphiVulg	0	0	1
Origanum vulgare	*Origanum vulgare* L.	OrigVulg	1	0	0
Oxalis acetosella	*Oxalis acetosella* L.	OxalAcet	1	0	0
Peucedanum palustre	*Peucedanum palustre* (L.) Moench	PeucPals	0	0	1
Phalaris arundinacea	*Phalaris arundinacea* L.	PhalArun	0	1	1
Phragmites australis	*Phragmites australis* (Cav.) Steud.	PhrgAust	0	0	1
Picea abies	*Picea abies* (L.) H. Karst.	PiceAbie	1	0	0
Pimpinella saxifraga	*Pimpinella saxifraga* L.	PimpSaxf	1	1	1
Pinus sylvestris	*Pinus sylvestris* L.	PinsSylv	1	1	1
Plantago coronopus	*Plantago coronopus* L.	PlanCorn	0	0	1
Plantago lanceolata	*Plantago lanceolata* L.	PlanLanc	0	1	1
Plantago major	*Plantago major* L. s. str.	PlanMajr	0	1	0
Plantago major spp. *winteri*	*Plantago major* spp. *winteri* (Wirtg.) W.Ludw.	PlanMajS	0	0	1
Plantago maritima	*Plantago maritima* L. s. l.	PlanMart	0	0	1
Platanthera bifolia	*Platanthera bifolia* (L.) Rich.	PlatBifl	0	1	0
Platanthera chlorantha	*Platanthera chlorantha* (Custer) Rchb.	PlatChlr	1	0	0

Poa annua	*Poa annua* L.	PoaAnnua	0	1	1
Poa compressa	*Poa compressa* L.	PoaCompr	1	1	1
Poa nemoralis	*Poa nemoralis* L.	PoaNemor	1	0	0
Poa pratensis agg.		PoaPrtAg	1	1	1
Poa remota	*Poa remota* Forselles	PoaRemot	1	0	0
Polygala vulgaris	*Polygala vulgaris* L. s. l.	PolgVulg	0	1	0
Polygonatum odoratum	*Polygonatum odoratum* (Mill.) Druce	PolgOdor	1	1	0
Polygonum aviculare	*Polygonum aviculare* L. (s. l.)	PolgAvic	0	0	1
Polygonum lapathifolium	*Polygonum lapathifolium* L.	PolgLapt	0	0	1
Polypodium vulgare	*Polypodium vulgare* L.	PolpVulg	1	1	1
Populus tremula	*Populus tremula* L.	PoplTrem	1	1	0
Potentilla anserina	*Potentilla anserina* L.	PotnAnsr	0	0	1
Potentilla argentea	*Potentilla argentea* L. s. str.	PotnArgn	0	1	1
Potentilla erecta	*Potentilla erecta* (L.) Raeusch.	PotnErec	1	1	0
Potentilla recta	*Potentilla recta* L.	PotnRect	0	1	0
Potentilla reptans	*Potentilla reptans* L.	PotnRept	1	1	0
Primula veris	*Primula veris* L.	PrimVers	1	1	0
Prunella vulgaris	*Prunella vulgaris* L.	PrunVulg	0	1	1
Prunus avium	*Prunus avium* (L.) L.	PrunAviu	1	1	1
Prunus padus	*Prunus padus* L.	PrunPads	1	0	0
Prunus spinosa	*Prunus spinosa* L. s. str.	PrunSpin	1	1	1
Pteridium aquilinum	*Pteridium aquilinum* (L.) Kuhn	PterAqui	1	1	0
Puccinellia capillaris	*Puccinellia capillaris* (Lilj.) Jansen	PuccCapl	0	0	1
Puccinellia distans	*Puccinellia distans* (Jacq.) Parl. s. str.	PuccDist	0	0	1
Quercus robur	*Quercus robur* L.	QuerRobr	1	1	0
Ranunculus repens	*Ranunculus repens* L.	RanuRepn	0	1	1
Rhinanthus angustifolius spp. *angustifolius*	*Rhinanthus angustifolius* spp. *angustifolius* C. C. Gmel	RhinAngS	0	0	1
Rhinanthus minor	*Rhinanthus minor* L.	RhinMinr	0	1	0
Ribes alpinum	*Ribes alpinum* L.	RibsAlpn	1	0	0
Rorippa palustris	*Rorippa palustris* (L.) Besser	RorpPals	0	0	1
Rosa canina agg.		RosCanAg	1	1	1
Rosa rugosa	*Rosa rugosa* Thunb.	RosaRugs	0	1	0
Rubus fruticosus agg.		RubFrtAg	1	1	1
Rubus idaeus	*Rubus idaeus* L.	RubsIdae	1	1	1
Rubus saxatilis	*Rubus saxatilis* L.	RubsSaxt	1	0	0
Rumex acetosa	*Rumex acetosa* L.	RumxAcet	1	1	1
Rumex acetosella	*Rumex acetosella* L. s. l.	RumxAcet	0	1	1
Rumex crispus	*Rumex crispus* L.	RumxCris	0	1	1
Sagina procumbens	*Sagina procumbens* L.	SagnProc	0	0	1
Salix aurita	*Salix aurita* L.	SalxAurt	1	0	0
Salix caprea	*Salix caprea* L.	SalxCapr	1	1	1
Samolus valerandi	*Samolus valerandi* L.	SamlValr	0	0	1
Sanicula europaea	*Sanicula europaea* L.	SancEurp	1	0	0
Saxifraga granulata	*Saxifraga granulata* L.	SaxfGran	0	1	0
Scleranthus annuus	*Scleranthus annuus* L. s. str.	SclrAnnu	0	0	1
Scrophularia nodosa	*Scrophularia nodosa* L.	ScrpNods	1	1	1
Scutellaria galericulata	*Scutellaria galericulata* L.	ScutGalr	0	0	1
Sedum acre	*Sedum acre* L.	SedmAcre	0	1	1
Sedum album	*Sedum album* L.	SedmAlbm	0	0	1
Sedum telephium agg.		SedTelAg	1	1	1
Selinum carvifolia	*Selinum carvifolia* (L.) L.	SelnCarv	0	1	1
Senecio sylvaticus	*Senecio sylvaticus* L.	SencSylv	0	1	1
Senecio vulgaris	*Senecio vulgaris* L.	SencVulg	0	1	1
Silene viscosa	*Silene viscosa* (L.) Pers.	SilnVisc	0	0	1
Silene vulgaris	*Silene vulgaris* (Moench) Garcke s. l.	SilnVulg	0	0	1
Sisymbrium altissimum	*Sisymbrium altissimum* L.	SismAlts	0	0	1
Solanum dulcamara	*Solanum dulcamara* L.	SolnDulc	0	0	1
Solidago virgaurea	*Solidago virgaurea* L.	SoldVirg	1	1	1
Sonchus arvensis	*Sonchus arvensis* L.	SoncArvn	0	0	1
Sorbus aucuparia	*Sorbus aucuparia* L.	SorbAucp	1	1	1

Sorbus intermedia	*Sorbus intermedia* (Ehrh.) Pers.	SorbIntr	1	1	0
Spergularia marina	*Spergularia marina* (L.) Besser	SperMarn	0	0	1
Spergularia rubra	*Spergularia rubra* (L.) J. Presl & C. Presl	SperRubr	0	1	0
Stellaria graminea	*Stellaria graminea* L.	StelGram	1	1	0
Stellaria holostea	*Stellaria holostea* L.	StelHols	0	1	0
Stellaria media	*Stellaria media* (L.) Vill. s. str.	StelMedi	0	0	1
Stellaria palustris	*Stellaria palustris* Hoffm.	StelPals	0	0	1
Tanacetum vulgare	*Tanacetum vulgare* L.	TancVulg	0	1	1
Taraxacum sect. *Ruderalia*		TarSecRd	0	1	1
Taxus baccata	*Taxus baccata* L.	TaxsBacc	1	0	0
Teesdalia nudicaulis	*Teesdalia nudicaulis* (L.) R. Br.	TeesNudc	0	1	1
Thlaspi arvense	*Thlaspi arvense* L.	ThlsArvn	0	0	1
Trientalis europaea	*Trientalis europaea* L.	TrieEurp	1	0	0
Trifolium arvense	*Trifolium arvense* L.	TrifArvn	0	1	0
Trifolium campestre	*Trifolium campestre* Schreb.	TrifCamp	0	1	0
Trifolium pratense	*Trifolium pratense* L.	TrifPrat	1	1	0
Trifolium repens	*Trifolium repens* L.	TrifRepn	0	1	0
Triglochin maritima	*Triglochin maritima* L.	TrigMart	0	0	1
Urtica dioica	*Urtica dioica* L. s. l.	UrtcDioi	0	1	1
Vaccinium myrtillus	*Vaccinium myrtillus* L.	VaccMyrt	1	1	0
Vaccinium vitis-idaea	*Vaccinium vitis-idaea* L.	VaccVits	1	1	0
Valeriana officinalis agg.	*Valeriana officinalis* L.	ValOffAg	0	1	1
Verbascum thapsus	*Verbascum thapsus* L.	VerbThap	0	1	1
Veronica arvensis	*Veronica arvensis* L.	VernArvn	0	1	1
Veronica chamaedrys	*Veronica chamaedrys* L. s. l.	VernCham	1	1	0
Veronica longifolia	*Veronica longifolia* L.	VernLong	0	0	1
Veronica officinalis	*Veronica officinalis* L.	VernOffc	1	1	1
Veronica serpyllifolia	*Veronica serpyllifolia* L.	VernSerp	0	1	1
Vicia cracca	*Vicia cracca* L. s. str.	ViciCrac	0	1	1
Vicia hirsuta	*Vicia hirsuta* (L.) Gray	ViciHirs	0	1	0
Vicia lathyroides	*Vicia lathyroides* L.	ViciLath	0	1	0
Vicia sativa	*Vicia sativa* L. s. str.	ViciSatv	0	1	0
Vicia sylvatica	*Vicia sylvatica* L.	ViciSylv	1	0	0
Vicia tetrasperma	*Vicia tetrasperma* (L.) Schreb.	ViciTetr	0	1	0
Vincetoxicum hirundinaria	*Vincetoxicum hirundinaria* Medik.	VincHirn	0	1	0
Viola canina	*Viola canina* L. s. str.	ViolCani	0	1	0
Viola riviniana	*Viola riviniana* Rchb.	ViolRivn	1	1	0
Viola tricolor	*Viola tricolor* L.	ViolTric	0	1	1

List includes species presence (1) and absence (0) data for the sampled habitats (C = coniferous forest, G = semi-natural grassland, S = rocky shore) and species abbreviations used in the ordination plots (S1 Fig).
