# Supplementary material for: New insights into island vegetation composition and species diversity—Consistent and conditional responses across contrasting insular habitats at the plot-scale
Source: PLoS One. 2018 Jul 6;13(7):e0200191. doi: 10.1371/journal.pone.0200191 (PMC6034865; doi:10.1371/journal.pone.0200191)
Supplement: S3 Table — (PDF) [file pone.0200191.s007.pdf]

S3 Table. Detailed description of environmental explanatory variables and applied methods.

Variable	Detailed description/ methods
Elevation (m.a.s.l.)	Plot height above the sea level.
Eastness	Converted easterly aspect of a plot, -1 = west / +1 = east [1].
Northness	Converted northerly aspect of a plot, - 1 = south and +1 = north [1].
Slope (°)	*In situ* estimates for plot slope, based on class means of slope categories defined by [2].
Skeletal fraction [a] (%)	Dried soil samples were scaled before and after sieving to obtain the proportion of the coarse fraction.
Soil depth (m)	Substrate depth to bedrock was extracted from soil depth maps (1:50,000) produced by the [3]. Only applied for the grassland and coniferous forest plots.<br>Depth to bedrock in the rocky shore plots was measured at three points at each site using a hand shovel and a tapeline.
Soil type	Soil type data for each plot were extracted from quaternary deposits maps (1:50,000) from SGU [3] and then dummy coded: glacial clay (TYPE_CL), sandy till and post-glacial sands (TYPE_SN), rock (TYPE_RO). Rock is defined as exposed bedrock with soil depth less than 50 cm. Soil type was not defined for the rocky shore plots.
Conductivity [a] (uS cm^{-1})	Soil conductivity was electrometrically measured from H_2O soil solution with the conductivity sensor of a pH meter (PCE-PHD 1).
Carbon-nitrogen ratio [a]	Organic soil carbon and total nitrogen were analyzed by dry combustions by using a TruSpec CN Analyzer (LECO CORPORATION, St. Joseph, Michigan), see [4].
Phosphorus [a] (mg kg^{-1})	Analyses of soil phosphorus were based on extraction with ammonium lactate-acetic acid and measures with the spectral photometer DR5000 UV / VIS (HACH-LANGE, Düsseldorf, Germany), see [4].
pH(H_2O) [a]	pH value based on H_2O soil solution extraction, determined with pH meter (PCE PHD 1, PCE HOLDING GMBH, Meschede, Germany) on unfiltered suspension.
Soil water	Weighted mean Ellenberg indicator value for moisture, see [5,6].
Site openness (%)	Three hemispherical fisheye photographs were taken at each site from the herb layer and converted into a binary structure. Binary images were analyzed with GAP LIGHT ANALYZER 2.0 [7] and the plot mean calculated. Site openness is the sky section uncovered by topographic features or vegetation as seen from the ground.
Grazing history index	A relative temporal estimate of management abandonment of grassland pastures along a continuous scale reflecting three regression phases. Based on the species indicator system defined by [8] for abandoned pastures and meadows in South Sweden. Only calculated for grassland plots.
Vegetated area (%)	Vegetated area, a proxy for the plant available area in rock crevices. Only applied for rocky shore plots.
Distance to mainland or large island (m)	Euclidian distance between a plot location and the nearest mainland or large island polygon (≥ 50 ha).
Proximity index	Considers the size and proximity distance of all islands or mainland polygons within a 500 m radius of the focal island polygon [9]. ArcGIS 10.2 (ESRI Inc., Redlands, California) extension V-LATE 2.0 [10].
Relative wave exposure index	Wind and wave exposure computed with WEMO 4.0 [11]. Calculations based on wind frequency and speed of eight compass directions and their fetch. Fetch was modified with a shoreline dataset and weighted with bathymetric data. REI values for rocky shore plots are based on single plot-specific values. REI values for grassland and coniferous forest plots are based on the arithmetic mean of the wave exposure values of all polygon vertex points of a sampled island (Table A4).
Habitat area (m²)	Based on aerial imagery evaluation (Swedish National Land Survey, [12] and *in situ* estimates of habitat cover and total island area.
Area (m²)	Total island area.
Rock cover (%)	In situ estimates of island area covered by exposed rock, using aerial images [12].
Tree_cover (%)	In situ estimates of island area covered by trees, using aerial images [12].

[a] Based on three composite soil samples within rooting depth at different positions within each plot. Samples were dried (60 °C) and sieved (mesh size < 2 mm) and an amount of 100 g of the fine substrate was retained for chemical analyses.

References

1. Zar JH. Biostatistical analysis. Prentice Hall; 1999.

2. AG Boden. Bodenkundliche Kartieranleitung mit 33 Abbildungen und 91 Tabellen. 4th ed. Bundesanstalt für Geowissenschaften und Rohstoffe, editor. Hannover: Schweizerbart; 1996.

3. Geological Survey of Sweden (SGU). Map generator: shore-level maps, bedrock maps, soil depth maps and quaternary deposits maps (1:50,000) [Internet]. 2015. Available: https://www.sgu.se/en/products/maps/map-generator/

4. Blume H-P, Stahr K, Leinweber P. Bodenkundliches Praktikum: Eine Einführung in pedologisches Arbeiten für Ökologen, insbesondere Land- und Forstwirte, und für Geowissenschaftler. Heidelberg: Spektrum Akademischer Verlag; 2011.

5. Ellenberg H, Weber, Heinrich, E., Dill R, Wirth V. Zeigerwerte von Pflanzen in Mitteleuropa. 3rd ed. Lehrstuhl f. Geobotanik d. Universität Göttingen, editor. Göttingen: Goltze; 2001.

6. Käfer J, Witte J-PM. Cover-weighted averaging of indicator values in vegetation analyses. J Veg Sci. 2004;15: 647–652.

7. Frazer GW, Canham CD, Lertzman KP. Gap Light Analyzer (GLA): imaging software to extract canopy structure and gap light transmission indices from true-colour fisheye photographs. Users manual and program documentation. Version 2.0. Simon Fraser University, British Columbia and Institute of Ecosystem Studies, New York; 1999.

8. Ekstam U, Forshed N. Om hävden upphör : kärlväxter som indikatorarter i ängs- och hagmarker = If grassland management ceases : vascular plants as indicator species in meadows and pastures. Solna: Statens naturvårdsverk; 1992.

9. Gustafson EJ, Parker GR. Using an index of habitat patch proximity for landscape design. Landsc Urban Plan. 1994;29: 117–130. doi:10.1016/0169-2046(94)90022-1

10. Lang S, Tiede D. vLATE Extension für ArcGIS: vektorbasiertes Tool zur quantitativen Landschaftsstrukturanalyse. ESRI Eur User Conf. 2003; 1–10.

11. Malhotra A, Fonseca MS. WEMo (Wave Exposure Model): formulation, procedures and validation. NOAA Tech Memo NOS NCCOS. 2007;65: 28.

12. Lantmäteriet. Map search and place-names [Internet]. 2013. Available: <https://kso.etjanster.lantmateriet.se> accessed 31.03.2015
