# Supplementary material for: New insights into island vegetation composition and species diversity—Consistent and conditional responses across contrasting insular habitats at the plot-scale
Source: PLoS One. 2018 Jul 6;13(7):e0200191. doi: 10.1371/journal.pone.0200191 (PMC6034865; doi:10.1371/journal.pone.0200191)
Supplement: S4 Table — Category A: species strongly decline in quantity or even die out shortly after management ceased. Category B: species possibly increase during an early phase, but decrease or go extinct in the medium term. Category C: species first increase during an early and intermediate phase, but decrease in a longer term. Regression phases: T1 = early phase; T2 = intermediate phase; T3 = late phase. † = extinct; -2 = strong decline; -1 = moderate decline; X = unchanged; +1 = moderate increase; +2 = strong increase. All possible combinations were ranked according to an increasing regression and projected on a numeric scale ranging from 0–100, representing grazing history values. (PDF) [file pone.0200191.s008.pdf]

S4 Table. **Defined combinations of temporal-quantitative changes and associated grazing history values of pasture indicator species after management abandonment, after [1].**

Category	Combination	T_1	T_2	T_3	Grazing history value
A	1	-2	†	†	0
	2	-1	†	†	3.125
	3	-2	-2	†	6.25
	4	-1	-2	†	9.375
	5	-1	-2	-2	12.5
	6	-1	-1	-2	15.625
B	7	X	†	†	18.75
	8	X	-2	†	21.875
	9	1	-2	†	25
	10	2	-2	†	28.125
	11	1	-1	†	31.25
	12	2	-1	†	34.375
	13	1	-2	-2	37.5
	14	2	-2	-2	40.625
	15	1	-1	-2	43.75
	16	X	-1	-2	46.875
	17	2	-1	-2	50
	18	X	-1	-1	53.125
	19	1	-1	-1	56.25
	20	2	-1	-1	59.375
C	21	1	X	-2	62.5
	22	2	X	-2	65.625
	23	X	1	-2	68.75
	24	1	1	-2	71.875
	25	2	1	-2	75
	26	1	2	-2	78.125
	27	2	2	-2	81.25
	28	1	X	-1	84.375
	29	2	X	-1	87.5
	30	1	1	-1	90.625
	31	2	1	-1	93.75
	32	1	2	-1	96.875
	33	2	2	-1	100

Category A: species strongly decline in quantity or even die out shortly after management ceased. Category B: species possibly increase during an early phase, but decrease or go extinct in the medium term. Category C: species first increase during an early and intermediate phase, but decrease in a longer term. Regression phases: T_1 = early phase; T_2 = intermediate phase; T_3 = late phase. † = extinct; -2 = strong decline; -1 = moderate decline; X = unchanged; +1 = moderate increase; +2 = strong increase. All possible combinations were ranked according to an increasing regression and projected on a numeric scale ranging from 0-100, representing grazing history values.

References

1. Ekstam U, Forshed N. Om hävden upphör : kärlväxter som indikatorarter i ängs- och hagmarker = If grassland management ceases : vascular plants as indicator species in meadows and pastures. Solna: Statens naturvårdsverk; 1992.
