# Supplementary material for: New insights into island vegetation composition and species diversity—Consistent and conditional responses across contrasting insular habitats at the plot-scale
Source: PLoS One. 2018 Jul 6;13(7):e0200191. doi: 10.1371/journal.pone.0200191 (PMC6034865; doi:10.1371/journal.pone.0200191)
Supplement: S5 Table — Calculated with the wave exposure model WEMO 4.0 [1] and ArcMap 10.2 (ESRI Inc., Redlands, California). REI = relative wave exposure index; SMHI = Swedish Meteorological and Hydrological Institute; IDW = inverse-distance-weighting. (PDF) [file pone.0200191.s009.pdf]

S5 Table. **Description of data requirements and step-wise calculation of REI values.**

Object	Description	Action	Tool / Function
	**Step I**<br>Data requirements and preparation<br>ArcMap 10.2		
Shoreline dataset	Shapefile containing the water area extracted from island vector database, generated from SPOT 5 satellite images (Lantmäteriet 2015, Saccess Sverige, <https://saccess.lantmateriet.se/portal/saccess_se.htm > accessed 31.03.2015).	Export of the seawater polygon from island database as a new shapefile.	ArcMap<br>Toolbar function<br>'Select Features'<br>Layers options<br>'Export data'
Bathymetry grid	Raster grid containing water depths with cell resolution 500 x 500 m from Baltic Sea Bathymetry Database, version 0.9.3. (Baltic Sea Hydrographic Commission 2013, <http://data.bshc.pro> accessed 20.06.2016).	Homogenization of grid data, definition of missing data.	ArcMap<br>Spatial Analyst Tool<br>'Raster Calculator'
Wind data	Wind speed and frequency for eight compass headings for the period 01.03.2010 – 01.03.2016. Surrogate data from observational stations closest to each study area (Karlskrona for Blekinge; Harstena for Västervik; Skarpö for Stockholm) (SMHI 2015, <http://opendata-download-metobs.smhi.se/explore> accessed 20.06.2016).	Calculation of mean wind speed and percent of wind frequency values for eight compass directions from hourly wind data.	
Point Dataset	Island polygon points from island database. Points must lie over the shoreline and bathymetry datasets, therefore originally recorded plot points could not be used.	Polygon vertices were added every 50 m along the polygon edges. Polygon vertices lie over shoreline and bathymetry data. Conversion of polygon vertices into point shapefile format (island polygon points).	Arc Map<br>Editing Tool<br>'Densify'<br>Data Management Tool 'Feature Vertices To Points'
	**Step II**<br>Calculation of REI values<br>WEMO 4.0, Relative Exposure Index Mode (REI mode) and ArcMap 10.2		
1. Settings	Project settings for REI calculations	Maximum fetch [a] limit = 10,000 m<br>Distance for bathymetry interrogation = 100 m<br>Use of powers for IDW functions checked.	WEMO 4.0<br>REI mode<br>'Settings'
2. Data import	Import of required datasets for REI calculations	Import of shoreline, bathymetry, point and wind datasets. Wind text file was created by manually entering mean wind speed and percent of wind frequency for eight compass directions for the period 01.03.2010 – 01.03.2016.	WEMO 4.0<br>REI mode<br>'Enter Wind Data'<br>'Add Data'<br>‚Select Point Dataset'
3. Running REI model	Calculation of unit-less site-specific REI values	Calculation of REI values for polygon points. Index values were automatically written to the attribute table of point shapefiles.	WEMO 4.0<br>REI mode<br>'Run model'
4. Generating plot-specific REI values	REI value of rocky shore plots	REI value from the closest island polygon point were written to the attribute table of the corresponding, plot point (located within the island polygon).	Arc Map<br>Analysis Tool<br>'Spatial Join'
	REI values of plots in the semi-natural grassland and coniferous forest	The arithmetic mean of REI values of all polygon points of an island were written to corresponding plot points of that island.	

Calculated with the wave exposure model WEMO 4.0 [1] and ArcMap 10.2 (ESRI Inc., Redlands, California). REI = relative wave exposure index; SMHI = Swedish Meteorological and Hydrological Institute; IDW= inverse-distance-weighting.

[a] Fetch is defined as the distance, which the wind can blow uninterrupted over water for a given compass direction, i.e. the distance from the location of interest to the first encountered land [2].

References

1. Malhotra A, Fonseca MS. WEMo (Wave Exposure Model): formulation, procedures and validation. NOAA Tech Memo NOS NCCOS. 2007;65: 28.

2. Pepper A, Puotinen ML. GREMO: a GIS-based generic model for estimating relative wave exposure. In: Andersen, R. S., Braddock RD, Newham LTH, editors. 18th World IMACS Congress and MODSIM09 International Congress on Modelling and Simulation. Cairns: Modelling and Simulation Society of Australia and New Zealand and International Association for Mathematics and Computers in Simulation; 2009. pp. 1964–1970. Available: http://ro.uow.edu.au/scipapers/3294/
