# Supplementary material for: New insights into island vegetation composition and species diversity—Consistent and conditional responses across contrasting insular habitats at the plot-scale
Source: PLoS One. 2018 Jul 6;13(7):e0200191. doi: 10.1371/journal.pone.0200191 (PMC6034865; doi:10.1371/journal.pone.0200191)
Supplement: S6 Table — Variables are explained in Table 2 and S3 Table. Region was treated as a separate set and is represented by factor levels. “[…]” = variable intercorrelated with variable in square brackets (r ≥ 0.6); ETV = explained total variation; “-” = variable not implemented; n.s. = not significant (p-value > 0.05); REGION_B = factor level Blekinge; REGION_S = factor level Stockholm. (PDF) [file pone.0200191.s010.pdf]

S6 Table. Summary statistics of CCA stepwise forward selection for defined variable-sets including information on collinear variables.

		Rocky shore		Semi-natural grassland		Coniferous forest	
Set	Variables	ETV (%)	p-value	ETV (%)	p-value	ETV (%)	p-value
Region	REGION_B	3.0	≤ 0.001	3.3	≤ 0.001	-	-
	REGION_S	1.5	≤ 0.001	-	-	3.9	≤ 0.001
Topography	ELEV	1.0	≤ 0.001	n.s.	n.s.	5.2	≤ 0.001
	EAST	n.s.	n.s.	n.s.	n.s.	1.8	0.006
	NOR	n.s.	n.s.	n.s.	n.s.	n.s.	n.s.
	SLO	0.7	0.001	n.s.	n.s.	n.s.	n.s.
Soil morphology	SKEL	0.8	≤ 0.001	1.7	0.013	1.3	0.030
	SOIL_D	0.6	0.004	[TYPE_SN]	[TYPE_SN]	2.3	0.001
	TYPE_SN	-	-	2.2	≤ 0.001	3.4	≤ 0.001
	TYPE_CL	-	-	-	-	6.0	≤ 0.001
Soil fertility	COND	0.9	≤ 0.001	[P]	[P]	[pH]	[pH]
	CN	0.9	≤ 0.001	3.1	≤ 0.001	2.7	≤ 0.001
	P	1.6	≤0.001	1.4	0.030	n.s.	n.s.
	PH	1.1	≤0.001	4.8	≤ 0.001	8.5	≤ 0.001
Soil water	EIV_M	3.3	≤ 0.001	2.7	≤ 0.001	4.8	≤ 0.001
Light availability	OPEN	2.8	≤ 0.001	2.9	≤ 0.001	7.9	≤ 0.001
Vegetated area	VEG_A	1.8	≤ 0.001	-	-	-	-
Grazing history	GHI	-	-	6.9	≤ 0.001	-	-
Distance	DMI	1.2	≤ 0.001	1.7	0.006	4.5	≤ 0.001
	PROX	n.s.	n.s.	2.9	0.001	n.s.	n.s.
	REI	1.2	≤ 0.001	2.1	0.002	n.s.	n.s.
Island configuration	HAB_A	0.6	0.016	2.5	≤ 0.001	[ISL_A]	[ISL_A]
	ISL_A	0.7	0.001	n.s.	n.s.	3.1	≤ 0.001
	R_COV	2.3	≤ 0.001	2.7	≤ 0.001	2.1	0.001
	T_COV	[R_COV]	[R_COV]	[R_COV]	[R_COV]	[R_COV]	[R_COV]

Variables are explained in Table 2 and S3 Table. Region was treated as a separate set and is represented by factor levels. “[…]“ = variable intercorrelated with variable in square brackets (r ≥ 0.6); ETV = explained total variation; “-“ = variable not implemented; n.s. = not significant (p-value > 0.05); REGION_B = factor level Blekinge; REGION_S = factor level Stockholm.
