# Supplementary material for: New insights into island vegetation composition and species diversity—Consistent and conditional responses across contrasting insular habitats at the plot-scale
Source: PLoS One. 2018 Jul 6;13(7):e0200191. doi: 10.1371/journal.pone.0200191 (PMC6034865; doi:10.1371/journal.pone.0200191)
Supplement: S7 Table — Gross effect is the variance explained when controlled for the factor region, net effect is the variance explained when controlled for all other variables, including region. Additionally, region was treated as a separate set. ETV = total explained variance; n.s = set without significant variables. (PDF) [file pone.0200191.s011.pdf]

S7 Table. Summary statistics of the pCCA series for the three habitat types, showing the relative contribution of each variable-set in explaining species composition.

Set	Gross effect (% of ETV)	Net effect (% of ETV)	p-value [a]
**Rocky shore**			
Region	4.49	2.73	≤ 0.001
Topography	1.66	0.95	≤ 0.001
Soil morphology	1.02	0.92	≤ 0.001
Soil fertility	4.26	2.24	≤ 0.001
Soil water	3.20	2.00	≤ 0.001
Light availability	2.51	0.83	≤ 0.001
Vegetated area	1.49	0.93	≤ 0.001
Distance	2.22	1.42	≤ 0.001
Island configuration	3.50	1.79	≤ 0.001
**Semi-natural grassland**			
Region	3.31	1.70	≤ 0.001
Topography	n.s.	n.s.	n.s.
Soil morphology	3.57	2.30	0.011
Soil fertility	8.51	5.18	≤ 0.001
Soil water	2.28	1.99	≤ 0.001
Light availability	2.37	1.17	0.040
Grazing history	6.87	4.32	≤ 0.001
Distance	5.82	4.18	≤ 0.001
Island configuration	4.89	2.32	0.010
**Coniferous forest**			
Region	3.85	1.82	≤ 0.001
Topography	6.99	2.44	≤ 0.001
Soil morphology	11.57	4.33	≤ 0.001
Soil fertility	9.96	3.96	≤ 0.001
Soil water	5.01	2.42	≤ 0.001
Light availability	7.41	2.13	≤ 0.001
Distance	3.69	2.90	≤ 0.001
Island configuration	5.08	2.94	≤ 0.001

Gross effect is the variance explained when controlled for the factor region, net effect is the variance explained when controlled for all other variables, including region. Additionally, region was treated as a separate set. ETV = total explained variance; n.s = set without significant variables. [a] p-values refer to net effects
