# Supplementary material for: New insights into island vegetation composition and species diversity—Consistent and conditional responses across contrasting insular habitats at the plot-scale
Source: PLoS One. 2018 Jul 6;13(7):e0200191. doi: 10.1371/journal.pone.0200191 (PMC6034865; doi:10.1371/journal.pone.0200191)
Supplement: S8 Table — For each habitat and proxy of species diversity, the most significant variables (fixed effects) and implemented random effects of the fitted minimal adequate models are shown. Corresponding variable-sets are presented for the significant variables. Variables are described in Table 2 and S3 Table. Std. Err. = Standard Error; df = degrees of freedom; ETV = explained total variance. (PDF) [file pone.0200191.s012.pdf]

S8 Table. Summary statistics of the linear mixed-effects models for species richness (SR) and species evenness (SE).

Habitat type	Proxy	Set	Variables	Estimate	Std. Err.	df	T-value	p-value	ETV (%)
Rocky shore	SR	Random effects	REGION, ISLAND ID						23.37
			Intercept	10.32	1.020	1.99	10.12	0.010	-
		Topography	ELEV	-0.73	0.180	269.04	-4.07	≤ 0.001	4.02
			SLO	-0.36	0.178	272.14	-2.04	0.042	2.00
		Soil morphology	SOIL_D	1.20	0.183	179.14	6.53	≤ 0.001	6.58
		Soil water	EIV_M	0.81	0.183	267.00	4.44	≤ 0.001	4.46
		Vegetated area	VEG_A	1.09	0.182	272.44	6.00	≤ 0.001	6.00
		Distance	PROX	0.38	0.182	177.82	2.08	0.039	2.08
			DMI	0.53	0.201	87.24	2.66	0.009	2.94
	SE	Random effects	REGION, ISLAND ID						20.51
			Intercept	0.603	0.017	1.92	35.18	0.001	-
		Soil fertility	P	-0.027	0.009	277.58	-3.00	0.004	5.31
		Light availability	OPEN [a]	0.020	0.010	240.33	1.93	0.055	3.83
		Vegetated area	VEG_A	-0.087	0.009	271.36	-9.71	≤ 0.001	16.94
Semi-natural grassland	SR	Random effects	REGION, ISLAND ID						14.46
			Intercept	10.77	0.760	0.91	14.18	0.056	-
		Soil morphology	TYPE_SN	1.32	0.374	93.10	3.53	≤ 0.001	10.07
		Soil fertility	pH	2.83	0.399	93.11	7.09	≤ 0.001	21.54
			P	-0.97	0.360	92.22	-2.70	0.008	7.38
			CN	0.83	0.401	83.87	2.07	0.042	6.31
		Grazing history	GHI	-2.11	0.398	91.91	-5.30	≤ 0.001	16.04
	SE	Random effects	REGION, ISLAND ID						8.19
			Intercept	0.26	0.007	29.82	36.66	≤ 0.001	-
		Soil fertility	pH	0.02	0.007	91.97	2.70	0.008	9.96
			CN [a]	0.01	0.007	74.11	1.98	0.051	7.06
		Grazing history	GHI	-0.04	0.007	81.79	-5.71	≤ 0.001	21.20
Coniferous forest	SR	Random effects	REGION, ISLAND ID						3.13
			Intercept	14.77	0.345	32.34	42.62	≤ 0.001	-
		Topography	ELEV	-0.77	0.356	93.83	-2.15	0.034	6.22
		Soil morphology	SOIL_D	1.10	0.336	100.90	3.27	0.002	8.92
			TYPE_CL	1.49	0.358	96.22	4.18	≤ 0.001	12.15
		Soil fertility	pH	3.29	0.425	93.53	7.74	≤ 0.001	26.74
			CN	-0.97	0.421	81.24	-2.31	0.023	7.91
		Distance	DMI	1.05	0.356	34.28	2.95	0.006	8.54
		Island configuration	R_COV	-0.77	0.371	38.36	-2.09	0.044	6.29
	SE	Random effects	REGION, ISLAND ID						22.09
			Intercept	0.24	0.005	35.68	48.12	≤ 0.001	-
		Soil fertility	pH	0.02	0.004	98.32	4.87	≤ 0.001	13.25
		Distance	DMI	0.01	0.005	36.76	2.50	0.017	9.24
		Island configuration	ISL_A	0.01	0.005	34.94	2.05	0.048	7.62

For each habitat and proxy of species diversity, the most significant variables (fixed effects) and implemented random effects of the fitted minimal adequate models are shown. Corresponding variable-sets are presented for the significant variables. Variables are described in Table 2 (main text) and Table in S3 Table. Std. Err. = Standard Error; df = degrees of freedom; ETV = explained total variance. [a] non-significant variables ($p > 0.05$) were later excluded from the variable-sets that participated in the partitioning procedures
