# Supplementary material for: New insights into island vegetation composition and species diversity—Consistent and conditional responses across contrasting insular habitats at the plot-scale
Source: PLoS One. 2018 Jul 6;13(7):e0200191. doi: 10.1371/journal.pone.0200191 (PMC6034865; doi:10.1371/journal.pone.0200191)
Supplement: S1 Text — (PDF) [file pone.0200191.s013.pdf]

S1 Text. How to calculate the grazing history index (GHI).

For each defined pasture indicator species [1], individual response numbers are assigned, corresponding to three defined regression phases (see S4 Table). Species response is defined as intra-specific change of species abundance during the regression process after management abandonment. The grazing history value of an indicator species on a numerical scale from 0 – 100 is a combination of the three response numbers corresponding to the regression phases. The grazing history index (GHI) is based on the cover-weighted average of the grazing history values of all indicator species present at a site. In practice, plot GHI reflects the probability of a site being recently managed (low GHI values) or that management ceased long time ago (high GHI values). For calculation of mean cover-weighted grazing history values (grazing history index) at least three indicator species must be present. Below this threshold, historic grazing by livestock cannot be detected with sufficient accuracy, i.e. these sites are treated as not being continuously pastured within the last decades (grazing history index = 101).

References

1. Ekstam U, Forshed N. Om hävden upphör : kärlväxter som indikatorarter i ängs- och hagmarker = If grassland management ceases : vascular plants as indicator species in meadows and pastures. Solna: Statens naturvårdsverk; 1992.
