# Supplementary material for: New insights into island vegetation composition and species diversity—Consistent and conditional responses across contrasting insular habitats at the plot-scale
Source: PLoS One. 2018 Jul 6;13(7):e0200191. doi: 10.1371/journal.pone.0200191 (PMC6034865; doi:10.1371/journal.pone.0200191)
Supplement: S2 Text — (PDF) [file pone.0200191.s014.pdf]

S2 Text. Gross and net effects on vegetation composition

We tested the effects of the variable-sets and examined their relative contributions on vegetation composition for each habitat type, by always treating the factor region as a covariable. These simple effects, not corrected for the effects of all other variable-sets can be referred to as gross effects [1]. Net effects, on the other hand, reveal the significance of the unique contribution of variable-sets [2] when controlled for all other sets, including region. CCA-based variance partitioning of vegetation composition in the habitat types was originally computed for both net and gross effects. The proportions of the relative contributions of gross and net effects in explaining vegetation composition in the habitats were found to be relatively similar, see S2 Fig. For reason of simplification and consistency with the parallel analyses of species diversity, only gross effects of variable-sets and their relative contributions were presented in the main text.

References

1. Harvolk S, Symmank L, Sundermeier A, Otte A, Donath TW. Human impact on plant biodiversity in functional floodplains of heavily modified rivers: a comparative study along German Federal Waterways. Ecol Eng. 2015;84: 463–475. doi:10.1016/j.ecoleng.2015.09.019

2. Økland RH, Eilertsen O. Canonical correspondence analysis with variation partitioning: some comments and an application. J Veg Sci. 1994; 117–126.
